# The effectiveness and safety of oral Chinese patent medicines in treating myocardial infarction complicated with heart failure: a network meta-analysis of 12 interventions

**DOI:** 10.3389/fcvm.2026.1832017

**Published:** 2026-05-21

**Authors:** Yiying Liu, Ruikang Liu, Kai Yang, Jun Li, Guancheng Ye, Xuanchun Huang, Shiyi Tao, Tiantian Xue, Yulian Yuan

**Affiliations:** 1 Department of Cardiology, Guang'anmen Hospital, China Academy of Chinese Medical Sciences, Beijing, China; 2 Graduate School of Beijing University of Chinese Medicine, Beijing, China; 3 Department of Rheumatology, Dongzhimen Hospital, Beijing University of Chinese Medicine, Beijing, China; 4 Department of Nephrology and Endocrinology, Yuquan Hospital, Tsinghua University, Beijing, China; 5 School of Medicine, Tsinghua University, Beijing, China

**Keywords:** heart failure, myocardial infarction, network meta-analysis, oral traditional Chinese patent medicine, randomized controlled trial

## Abstract

**Objective:**

To systematically evaluate the effectiveness of different oral Chinese patent medicines (CPMs) combined with conventional treatment (CT) in the treatment of myocardial infarction with heart failure (MI-HF) using a Bayesian network Meta-analysis.

**Methods:**

Computerized searches were conducted in CNKI, WanFang, VIP, SinoMed, PubMed, EMbase, Web of Science, and the Cochrane Library for relevant randomized controlled trials (RCTs) published from database inception until October 15, 2025. The quality of the included studies was assessed using the Cochrane Risk of Bias tool (RoB2). Statistical analysis was performed using Stata 17.0 and RevMan 5.3.

**Results:**

A total of 42 RCTs were included, involving 12 oral CPMs, 6 outcome indicators and adverse reactions. In terms of improving the total clinical effective rate, Yixinshu capsules demonstrated the best therapeutic effect. Xintong oral liquid showed the best effect in improving LVEF and reducing LVESD. For reducing LVEDD, Shexiang Baoxin pill was optimal. Guanxin Shutong capsules were the most effective for lowering NT-proBNP and increasing the 6 MWT distance.

**Conclusion:**

Oral CPMs combined with CT significantly improve cardiac function, ventricular remodeling, and exercise tolerance in MI-HF. While short-term tolerability is favorable, poor reporting limits definitive safety conclusions. Notably, Xintong Oral Liquid, Guanxin Shutong Capsules, and Qili Qiangxin Capsules exhibit the most outstanding comprehensive benefits.

**Systematic Review Registration:**

https://www.crd.york.ac.uk/PROSPERO/, PROSPERO CRD420251234570.

## 1 Introduction

Myocardial Infarction (MI) is a necrotic myocardial lesion caused by acute, persistent coronary ischemia and hypoxia, representing one of the leading cardiovascular diseases responsible for global morbidity and mortality (1). Extensive myocardial necrosis severely compromises cardiac pumping function, with massive cardiomyocyte death and subsequent fibrotic repair triggering left ventricular remodeling and substantially increasing the risk of heart failure (HF) (2). When both conditions coexist (MI-HF), the condition is characterized by ventricular remodeling and severe impairment of myocardial contractile and diastolic functions. Patients typically present with dyspnea, fluid retention, markedly reduced exercise tolerance, and profoundly compromised quality of life (3, 4). MI-HF patients face significantly higher cumulative 5-year risks of all-cause mortality (36.3% vs. 10.1%) and cardiovascular mortality (19.1% vs. 3.3%) within the first year following discharge compared with MI patients alone (5, 6). Early identification of patients at risk for cardiogenic shock, a life-threatening complication frequently seen in the MI-HF continuum, is crucial for improving outcomes. Artificial intelligence-based prediction models have recently been systematically reviewed for their ability to forecast age-related cardiogenic shock, offering a promising tool for risk stratification and personalized management in this vulnerable population (7).

Currently, research on the prevention and treatment of MI-HF using Traditional Chinese Medicine (TCM) has achieved considerable progress. Oral Chinese patent medicines (CPMs), distinguished by their convenient administration and advantages of multi-component, multi-target holistic regulation, demonstrate considerable potential in improving cardiac function, alleviating clinical symptoms, and enhancing quality of life in MI-HF patients (8). This multicomponent, multitarget paradigm aligns closely with the concept of natural product-based therapeutics. A recent narrative review has summarized the current evidence on natural products and their bioactive ingredients in the treatment of cardiovascular aging (9), a key pathological process that underlies the progression from myocardial infarction to heart failure. These insights further support the rationale for developing natural-derived medicines, including certain Chinese patent medicines, for age-related cardiovascular conditions such as MI-HF. However, existing studies examining oral CPMs for MI-HF are generally of suboptimal quality, with some randomized controlled trials (RCTs) employing small sample sizes and methodologically flawed designs. Notably, direct comparative analyses of efficacy between different CPMs remain scarce (10).

In recent years, network Meta-analyses examining CPMs for cardiovascular diseases have proliferated. Wang Aolong (11) compared the efficacy of various Chinese patent medicines for coronary heart disease complicated with HF, while Yuan Guoqiang (12) reviewed advances in Meta-analyses of CPMs for acute MI, thereby providing evidence-based guidance for selecting specific medications for particular disease types. Zhang Chidao (13) conducted an evidence mapping analysis of clinical studies on Chinese patent medicines for post-acute MI HF, systematically delineating the research landscape and evidence distribution of MI-HF; however, this work did not quantitatively compare or rank the relative efficacy of various CPMs. Existing systematic reviews have predominantly focused on single CPMs, specific pharmaceutical ingredients, or single disease entities (either HF or MI in isolation). To date, no network Meta-analysis specifically targeting the MI-HF population has systematically compared the efficacy of multiple oral CPMs.

Network Meta-analysis enables the simultaneous integration of both direct and indirect comparative evidence, facilitating quantitative comparison and ranking of the efficacy and safety of different therapeutic regimens within the same evidence network (14). This study employs network Meta-analysis methodology to systematically evaluate RCTs examining various oral CPMs combined with conventional treatment (CT) for MI-HF, investigating the differential effects of oral CPMs in improving total clinical effective rate, cardiac function, cardiac structure, and quality of life, thereby providing evidence-based support for optimizing integrated TCM treatment strategies for MI-HF.

## 2 Materials and methods

### 2.1 Search strategy

A comprehensive computerized literature search was conducted across eight databases: China National Knowledge Infrastructure (CNKI), VIP Chinese Scientific Journals Database (VIP), WanFang Academic Journals Database (WanFang), Chinese Biomedical Literature Service System (SinoMed), Cochrane Library, PubMed, EMbase, and Web of Science. The search covered the period from database inception to October 15, 2025, targeting RCTs investigating treatments for MI-HF, with language restrictions limited to Chinese and English. Search terms included Medicine, Chinese Traditional, Myocardial Infarction, Heart Failure, and associated keywords. Literature management software was employed for deduplication and cross-verification of search results to enhance retrieval comprehensiveness and prevent duplicate counting. Detailed search strategies for each database are provided in Supplementary Appendix S1. The study selection process adhered to the Preferred Reporting Items for Systematic Reviews and Meta-Analyses (PRISMA) guidelines (15), and the meta-analysis protocol was prospectively registered with the International Prospective Register of Systematic Reviews (PROSPERO, NO. CRD420251234570).

### 2.2 Inclusion and exclusion criteria

#### 2.2.1 Participants

Patients of any sex, age, or disease duration were eligible, provided they met the diagnostic criteria for MI complicated with HF (16): (1) documented history of MI or imaging evidence and (2) presence of symptoms and/or signs of HF, confirmed by echocardiographic evidence of structural and functional cardiac abnormalities.

#### 2.2.2 Interventions

The control group received CT in accordance with clinical guidelines. To accurately reflect variations in therapeutic intensity and drug classes, CT regimens were classified into four distinct categories: (1) Standard conventional treatment (SCT), defined as classic guideline-directed medical therapy primarily comprising antiplatelet agents, angiotensin-converting enzyme inhibitors/angiotensin II receptor blockers (ACEI/ARB), beta-blockers, mineralocorticoid receptor antagonists, statins, and diuretics, alongside routine adjuncts such as oral nitrates or anticoagulants. (2) ARNI-based therapy, which incorporated modern sacubitril/valsartan into the standard regimen. (3) Intravenous vasoactive agents (IVA) therapy, involving intensive intravenous medications (e.g., rhBNP, levosimendan, or milrinone) typically required for acute decompensation. (4) Enhanced external counterpulsation (EECP) therapy, which integrated SCT with device-assisted hemodynamic support. One study employed a double-blind design using a placebo simulation. The experimental group received CT combined with one specific oral CPM. Studies employing a placebo alone in lieu of CT were excluded to satisfy ethical requirements for MI-HF treatment.

#### 2.2.3 Study design

RCTs meeting the connectivity criteria for network meta-analysis were included, requiring that all interventions formed a connected network through common comparator arms to ensure feasibility of indirect comparisons.

#### 2.2.4 Outcome measures

Primary outcomes included: (1) total clinical effective rate, (2) left ventricular ejection fraction (LVEF), (3) left ventricular end-diastolic diameter (LVEDD), (4) left ventricular end-systolic diameter (LVESD), (5) N-terminal pro-B-type natriuretic peptide (NT-proBNP) levels, and (6) 6-minute walk test (6 MWT) distance. Additionally, adverse events reported in the included literature were systematically collected and subjected to descriptive analysis to evaluate the safety of each treatment regimen.

### 2.3 Exclusion criteria

Studies meeting any of the following conditions were excluded: (1) insufficient sample size (total sample size fewer than 30 participants), (2) theoretical studies, basic experimental research, retrospective studies, reviews, systematic reviews, or meta-analyses, (3) interventions involving non-oral CPMs, or reports based on expert experience or case reports, (4) study designs not conforming to randomized controlled principles, and (5) duplicate publications, with only the version having the largest sample size or most complete information retained.

### 2.4 Study selection and data extraction

Data Extraction and Quality Assessment were performed independently by two reviewers (Liu YY and Liu RK), with discrepancies resolved by a third reviewer (Ye GC). Data were extracted and subsequently cross-verified. In instances of discrepancies, a third researcher was consulted to provide adjudication. The following information was collected from each article: the name of the first author, age, gender, the year of disease onset as reported in the article, research design, and outcome indicator values. All data are derived from the final outcome indicators. The risk of bias in RCTs was assessed using the RCT bias risk assessment tool recommended in the Cochrane Handbook version 5.1.0 (17).

### 2.5 Risk of bias assessment

The Cochrane Risk of Bias 2 (RoB 2) tool was employed to evaluate the risk of bias in included studies. The assessment covered the following six domains: (1) bias arising from the randomization process, (2) bias due to deviations from intended interventions, (3) bias in measurement of the outcome, (4) bias due to missing outcome data, (5) bias in selection of the reported result, and (6) overall risk of bias. Each domain was judged as “low risk”, “some concerns”, or “high risk” based on specific information reported in the source literature.

### 2.6 Statistical analysis

This study employed network meta-analysis to integrate direct and indirect comparative evidence, conducting quantitative comparisons of the efficacy of 12 oral CPMs combined with conventional treatment for MI-HF. Continuous variables were expressed as mean ± standard deviation, and dichotomous variables were presented as frequencies. Statistical analyses were performed using Stata version 17.0, with the mvmeta package used to execute the network meta-analysis and the network package employed for network graph plotting. Literature quality and risk of bias assessments were conducted using Review Manager 5.3. All effect sizes were reported with 95% confidence intervals. The significance level was set at α = 0.05. Heterogeneity was assessed using the *I*^2^ statistic; *I*^2^ ≤ 50% and *P* ≥ 0.1 indicated low heterogeneity, for which a fixed-effects model was applied; otherwise, a random-effects model was utilized. When substantial heterogeneity was identified (*I*^2^ > 50% or *P* < 0.1), subgroup analyses were conducted to explore potential sources of heterogeneity. Additionally, sensitivity analyses were performed by sequentially excluding studies with high risk of bias or subgroups exhibiting significant heterogeneity to assess the robustness of the findings. The surface under the cumulative ranking curve (SUCRA) was used to rank the probabilities of intervention efficacy; higher SUCRA values indicated a greater probability that the intervention was the optimal treatment. The evidence network constructed in this study consisted exclusively of indirect comparisons, with all nodes derived from two-arm trials, resulting in no closed loops. According to methodological guidelines, inconsistency testing was not required under these circumstances.

## 3 Results

### 3.1 Characteristics of included studies

**Figure 1:**
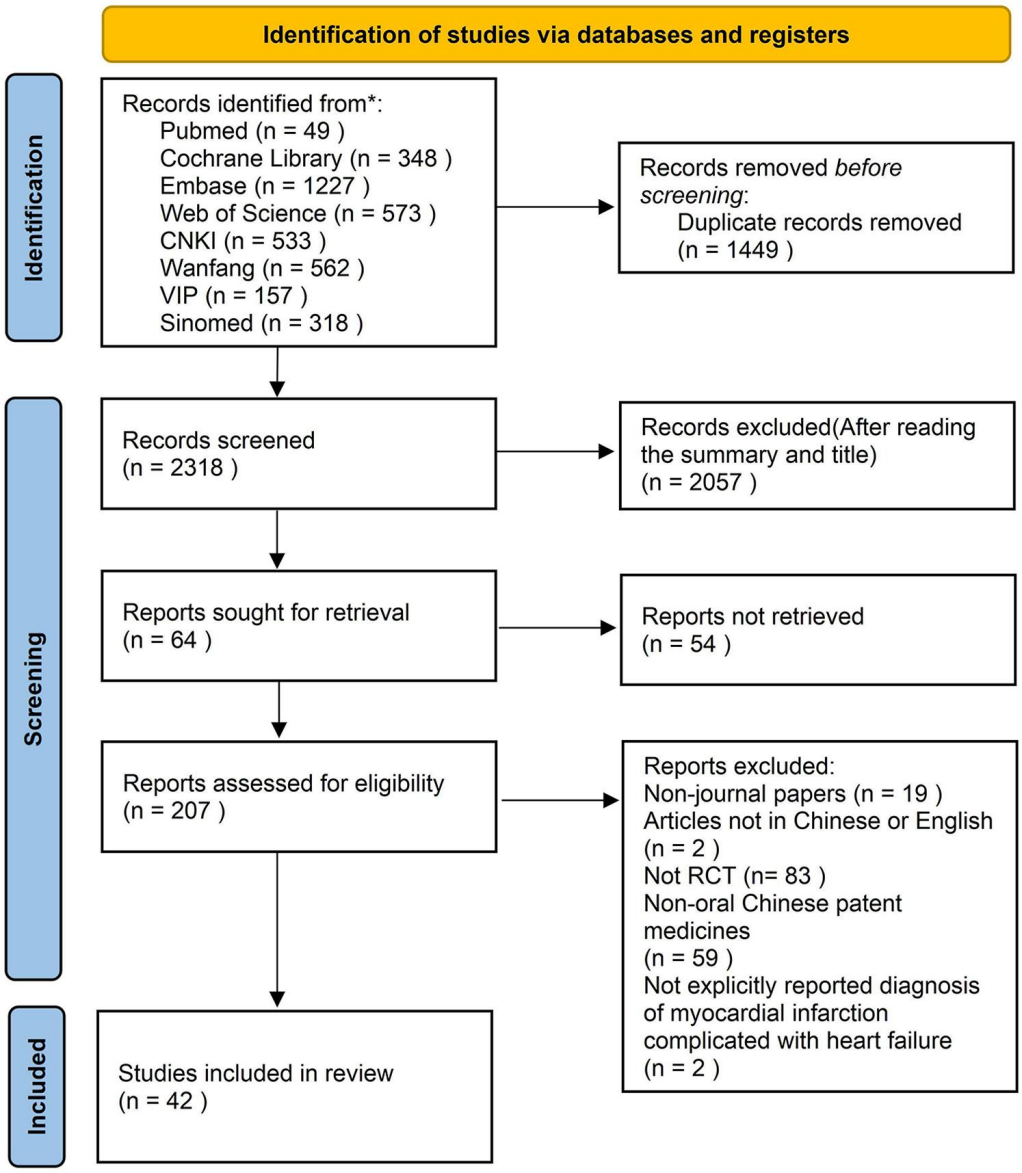
Document screening process and results.

A total of 3,767 records were identified through database searching. After deduplication (*n* = 1,449), 2,111 records were excluded by screening titles and abstracts. The remaining 207 articles were assessed for full-text eligibility, of which 165 were excluded for not meeting the inclusion criteria (non-RCTs or other reasons). Finally, 42 RCTs were included in the present meta-analysis (18–59). The study selection flow diagram is presented in Figure 1.

### 3.2 TCM syndrome characteristics of the included studies

Among the 42 included RCTs, only four explicitly specified TCM syndromes as inclusion criteria: Blood stasis in the heart (33), Qi deficiency with intermingled phlegm and blood stasis (41), Qi deficiency and blood stasis (42), and Yang deficiency with water retention (57). The remaining 38 studies did not restrict patient enrollment based on specific TCM syndrome differentiation. However, a review of the theoretical rationales provided in the original literature revealed a broad consensus that the core TCM pathogenesis of MI-HF belongs to deficiency in origin and excess in superficiality. Specifically, Qi stagnation and blood stasis was identified as the predominant syndrome pattern by the majority of the studies, while “Liver and kidney deficiency” was also noted (52).

### 3.3 Classification of study characteristics

A total of 42 RCTs (18–59) were included in this study (Supplementary Table S1), involving 12 oral CPMs, including Qili Qiangxin Capsules, Compound Danshen Dripping Pills, Shexiang Baoxin Pills, Yixinshu Capsules, Xuefu Zhuyu Capsules, Xintong Oral Liquid, Xinnaoshutong Capsules, Tongxinluo Capsules, Shexiang Tongxin Dripping Pills, Qishen Yiqi Dripping Pills, Huangqi Baoxin Granules, and Guanxinshutong Capsules (Supplementary Table S2). Baseline characteristics were comparable between the two groups in all included studies.

### 3.4 Risk of bias assessment results

**Figure 2:**
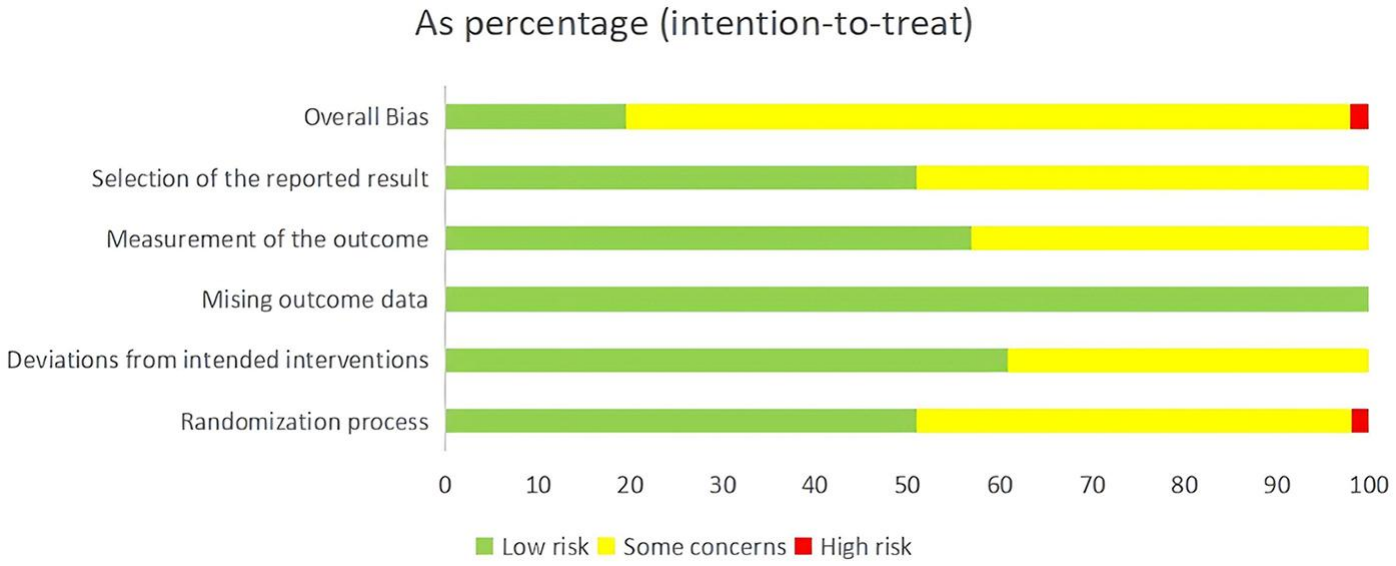
Risk of bias assessment included in the study.

Risk of bias was assessed for all included studies. Regarding bias arising from the randomization process, all included RCTs mentioned random allocation. Among them, 18 studies (18, 22, 24, 25, 32, 33, 38–43, 47, 49–51, 55, 57) used the random number table method and one study (23) used the random permutation method; these were rated as low risk. 1 study (58) used admission sequence for randomization and was rated as high risk. 1 study (41) adopted a double-blind design and was rated as low risk. The remaining studies did not report blinding or allocation concealment methods and were rated as unclear. All studies had complete outcome data with no other detected biases, and were therefore rated as low risk for these domains. Due to the inability to determine whether selective outcome reporting was present, this domain was rated as unclear for all studies. The risk of bias assessment results for the included studies are presented in Figure 2.

### 3.5 Evidence network

**Figure 3:**
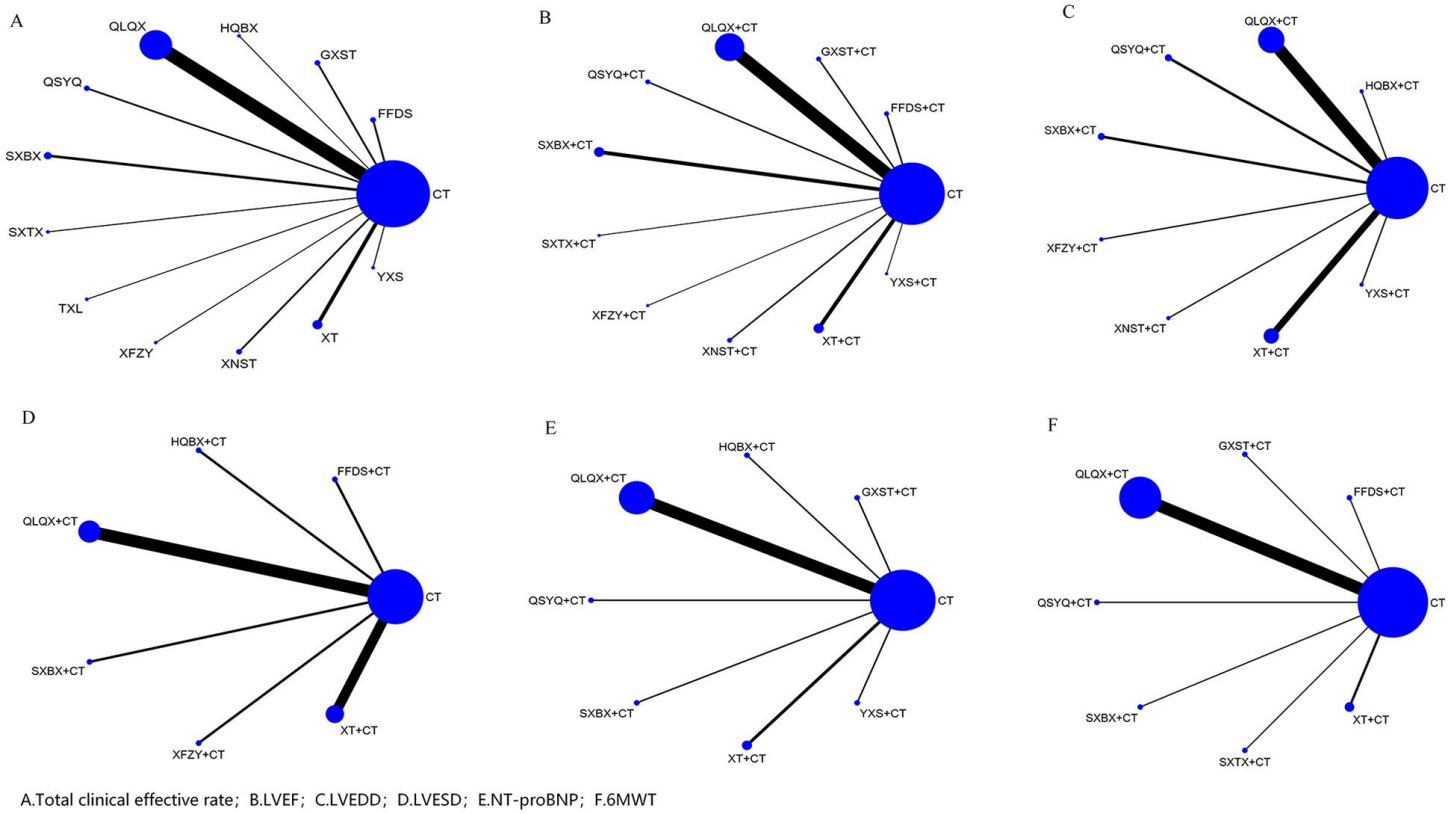
Evidence network of each outcome indicator. **(A)** Total clinical effective rate, **(B)** LVEF, **(C)** LVEDD, **(D)** LVESD, **(E)** NT-proBNP, **(F)** 6 MWT.

Figure 3 presents the evidence network of 12 CPMs combined with CT for treating MI-HF. In the network diagram, each node represents a treatment regimen; the node size is proportional to the number of participants, and the thickness of the connecting lines between nodes reflects the amount of direct comparison evidence available between different treatment regimens. This study lacked direct comparison evidence between the various interventions, and the evidence network structure did not form closed loops; therefore, inconsistency testing was not performed.

### 3.6 Total clinical effective rate

34 RCTs (18–24, 26–28, 30–32, 35–40, 42, 43, 45–51, 53–59)reported the total clinical effective rate, involving 12 CPMs. Compared to CT alone, combining CT with YXS [RR = 1.35, 95% CI (1.06, 1.72)], TXL [RR = 1.37, 95% CI (1.01, 1.86)], SXBX [RR = 1.26, 95% CI (1.13, 1.40)], XNST [RR = 1.25, 95% CI (1.09, 1.44)], FFDS [RR = 1.22, 95% CI (1.06, 1.39)], XT [RR = 1.20, 95% CI (1.10, 1.30)], QLQX [RR = 1.18, 95% CI (1.13, 1.24)], or GXST [RR = 1.18, 95% CI (1.05, 1.33)] significantly improved the total clinical effective rate (Supplementary Table S3).

**Figure 4:**
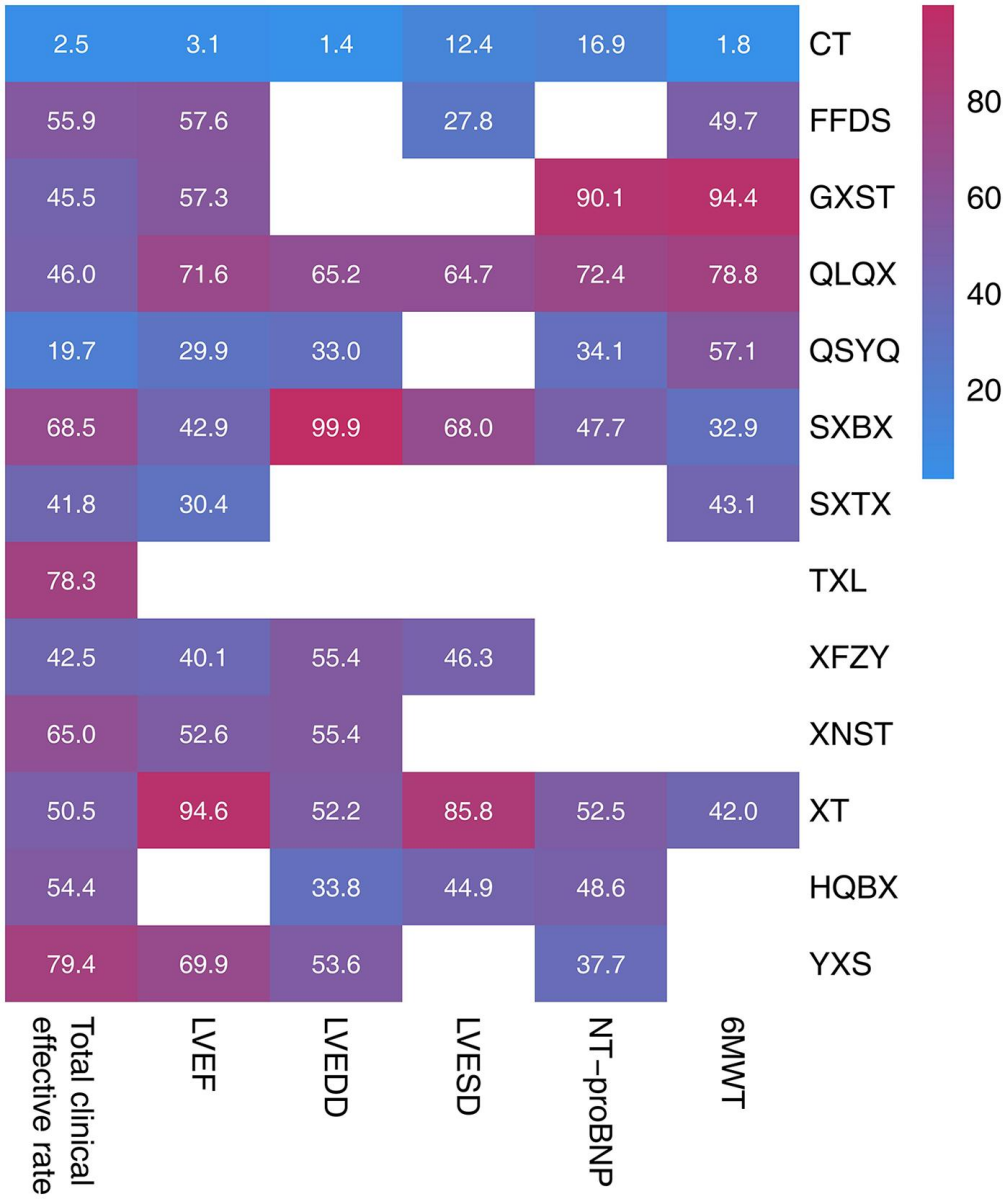
Heat map of SUCRA values for various intervention measures.

**Figure 5:**
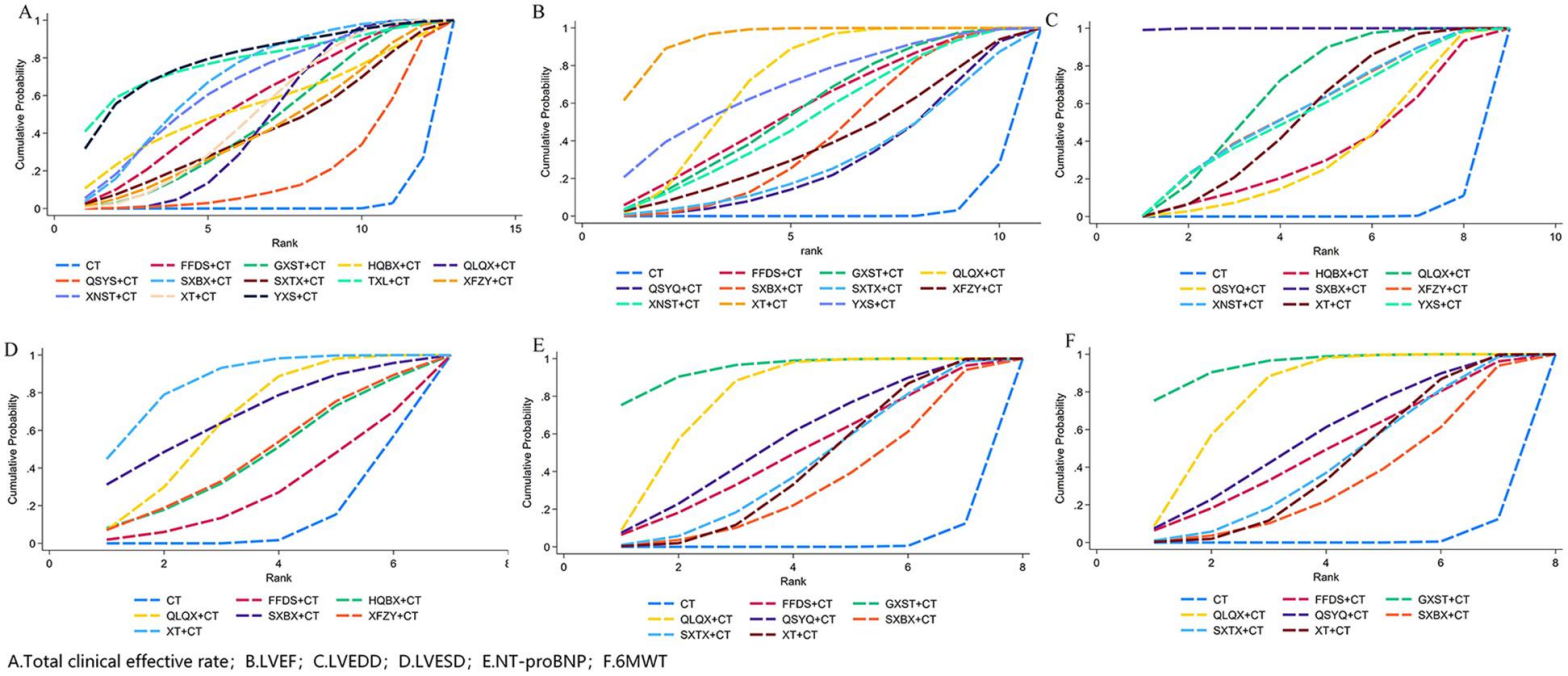
Ranking probabilities (SUCRA) for each outcome. **(A)** Total clinical effective rate, **(B)** LVEF, **(C)** LVEDD, **(D)** LVESD, **(E)** NT-proBNP, **(F)** 6 MWT.

We performed pairwise comparisons using meta-analysis. Adjunctive CPMs significantly improved overall clinical efficacy, yielding highly robust results without heterogeneity [RR = 1.20, 95% CI (1.17, 1.24), *P* < 0.001, *I*^2^ = 0%; Supplementary Figure S1]. According to the SUCRA values, the probability ranking of the total clinical effective rate for the 12 CPMs in treating MI-HF, from highest to lowest, was as follows: YXS (79.4%), followed by TXL (78.3%), SXBX (68.5%). The SUCRA values are detailed in Figures 4, 5, Table 1 and Supplementary Figures S2–S7.

**Table 1:** The SUCRA values of each outcome.

Intervention	Total clinical effective rate	Rank	LVEF	Rank	LVEDD	Rank	LVESD	Rank	NT-proBNP	Rank	6 MWT	Rank
CT	2.5	13	3.1	11	1.4	9	12.4	7	16.9	8	1.8	8
FFDS	55.9	5	57.6	4	–	–	27.8	6	–	–	49.7	4
GXST	45.5	9	57.3	5	–	–	–	–	90.1	1	94.4	1
QLQX	46	8	71.6	2	65.2	2	64.7	3	72.4	2	78.8	2
QSYQ	19.7	12	29.9	10	33.0	8	–	–	34.1	7	57.1	3
SXBX	68.5	3	42.9	7	99.9	1	68.0	2	47.7	5	32.9	7
SXTX	41.8	11	30.4	9	–	–	–	–	–	–	43.1	5
TXL	78.3	2	–		–	–	–	–	–	–	–	–
XFZY	42.5	10	40.1	8	55.4	4	46.3	4	–	–	–	–
XNST	65	4	52.6	6	55.4	3	–	–	–	–	–	–
XT	50.5	7	94.6	1	52.2	6	85.8	1	52.5	3	42	6
HQBX	54.4	6	–	–	33.8	7	44.9	5	48.6	4	–	–
YXS	79.4	1	69.9	3	53.6	5	–	–	37.7	6	–	–

### 3.7 LVEF

38 RCTs (18–23, 25, 27–34, 36–47, 49–59) reported LVEF, involving 10 CPMs. The results of the network meta-analysis showed that XT [MD = 9.26, 95% CI (6.83, 11.70)], QLQX [MD = 6.66, 95% CI (5.26, 8.06)], YXS [MD = 7.00, 95% CI (1.48, 12.52)], FFDS [MD = 5.65, 95% CI (1.04, 10.27)], GXST [MD = 5.61, 95% CI (1.85, 9.38)], XNST [MD = 5.23, 95% CI (1.11, 9.36)], and SXBX [MD = 4.52, 95% CI (1.77, 7.27)] combined with CT were superior to CT alone in improving LVEF. The confidence interval of the network meta-analysis result for QSYQ crossed the line of null effect [MD = 3.08, 95% CI (−1.25, 7.40)], indicating no statistically significant difference compared to CT (Supplementary Table S4).

Meta-analysis showed that adjunctive CPMs significantly improved LVEF compared to CT alone [WMD = 6.26, 95% CI (5.32, 7.20), *P* < 0.001, *I*^2^ = 86.5%] (Supplementary Figure S8). To explore the sources of heterogeneity and validate the robustness of the results, we conducted comprehensive subgroup analyses across different dimensions. Subgroup analysis (Supplementary Figure S9) confirmed significant LVEF improvements across the SCT [WMD = 6.18, 95% CI (5.05, 7.31)], IVA [WMD = 7.24, 95% CI (5.40, 9.09)], and EECP [WMD = 5.84, 95% CI (3.62, 8.07)] regimens, with additive benefits remaining robust even under modern ARNI-based therapy [WMD = 3.72, 95% CI (0.35, 7.10), *P* = 0.031]. Subgroup analysis confirmed significant LVEF improvements across all treatment durations (3–360 days, Supplementary Figure S10), with the 14-day subgroup effectively eliminating heterogeneity [WMD = 5.31, 95% CI (4.50, 6.12), *I*^2^ = 0.0%]. Subgroup analyses further confirmed consistent LVEF benefits across all MI stages (Supplementary Figure S11). Stratification by CPM category (Supplementary Figure S12) aligned with the network meta-analysis, with all formulas yielding significant improvements (*P* < 0.05), XT exhibited the largest effect size [WMD = 9.27, 95% CI (5.18, 13.35)].

Based on the SUCRA analysis, combination therapy with XT was the most effective intervention for improving LVEF values in patients with MI-HF, with a SUCRA value of 94.6%, followed by QLQX (71.6%).

### 3.8 LVEDD

23 RCTs (21, 24–26, 29, 31, 34, 35, 37–43, 45–47, 49, 54–56, 59) reported LVEDD, involving 8 types of CPMs. The results of the network meta-analysis showed that SXBX [MD = −15.32, 95% CI (−19.29, −11.34)], QLQX [MD = −6.65, 95% CI (−8.41, −4.89)], XFZY [MD = −6.12, 95% CI (−11.14, −1.10)], XNST [MD = −6.10, 95% CI (−11.15, −1.05)], YXS [MD = −6.00, 95% CI (−11.34, −0.66)], XT [MD = −5.78, 95% CI (−8.17, −3.39)], and QSYQ [MD = −4.00, 95% CI (−7.78, −0.23)] were superior to CT in reducing LVEDD. The therapeutic advantage of HQBX was not significant [MD = −3.93, 95% CI (−9.15, 1.29)], suggesting no definitive benefit within the indirect comparison framework (Supplementary Table S5).

The results of the meta-analysis were consistent with the network meta-analysis findings [MD = −6.84, 95% CI (−8.07, −5.60), *P* < 0.001, *I*^2^ = 90.3%]. Leave-one-out sensitivity analysis did not reveal the source of heterogeneity. Subgroup analysis by CPMs category showed that all 8 medicines had statistically significant effects, among which SXBX exhibited the largest effect size (MD = −13.22), while HQBX [MD = −3.93, 95% CI (−5.91, −1.95)] showed no statistical significance (Supplementary Figure S13). The reduction in LVEDD remained highly significant across all background therapies, including the modern ARNI-based subgroup, which notably demonstrated a complete elimination of within-group heterogeneity (Supplementary Figure S14).

Based on the SUCRA analysis, combination therapy with SXBX was the most effective intervention for improving LVEDD in patients, with a SUCRA value as high as 99.9%, followed by QLQX (65.2%) and XNST (55.4%).

### 3.9 LVESD

13 RCTs (19, 21, 24, 34, 38–40, 42, 46, 47, 49, 54, 59) reported LVESD, involving 6 types of CPMs. The results of the network meta-analysis showed that XT [MD = −7.26, 95% CI (−10.50, −4.02)] and QLQX [MD = −5.12, 95% CI (−8.04, −2.20)] combined with CT demonstrated significant advantages in improving LVESD. However, the confidence interval of the network meta-analysis result for FFDS crossed the line of null effect [MD = −1.28, 95% CI (−7.56, 5.00)], indicating no statistically significant difference compared to CT (Supplementary Table S6).

Meta-analysis showed that adjunctive CPMs significantly reduced LVESD compared to CT alone [WMD = −5.20, 95% CI (−6.79, −3.60), *P* < 0.001, *I*^2^ = 95.2%]. Leave-one-out sensitivity analysis did not conclusively reveal the source of heterogeneity. Subgroup analysis according to CT confirmed that the consistent reduction of LVESD was maintained across SCT, ARNI-enhanced subgroups, and IVA (Supplementary Figure S15). Furthermore, subgroup analysis by CPMs category corroborated the network meta-analysis, confirming their efficacy (Supplementary Figure S16).

Based on the SUCRA analysis, combination therapy with XT was the most effective intervention for improving LVESD in patients, with a SUCRA value of 85.8%, followed by SXBX (68.0%) and QLQX (64.7%).

### 3.10 NT-proBNP

16 RCTs (18, 23, 27, 32, 33, 39, 41, 44, 48–50, 52, 53, 55, 56, 59) reported NT-proBNP, involving 7 types of CPMs. According to the network meta-analysis, compared with CT, GXST + CT [MD = −787.82, 95% CI (−1,402.83, −172.81)] and QLQX + CT [MD = −365.75, 95% CI (−586.58, −144.92)] both demonstrated statistically significant efficacy in reducing NT-proBNP levels. However, the efficacy of XT + CT, HQBX + CT, SXBX + CT, YXS + CT, and QSYQ + CT did not meet the threshold for statistical significance, suggesting that the clinical effects of these combination treatment regimens still require further validation (Supplementary Table S7).

Meta-analysis revealed that adjunctive CPMs significantly enhanced NT-proBNP reduction compared to CT alone [MD = −27.21, 95% CI (−31.38, −23.04), *P* < 0.001, *I*^2^ = 99.4%]. Leave-one-out sensitivity analysis did not reveal the source of heterogeneity. Stratification by CPMs category showed that the GXST exhibited the most significant effect size [MD = −787.82, 95% CI (−881.37, −694.27)], XT demonstrated prominent efficacy [MD = −311.33, 95% CI (−436.70, −185.97)] (Supplementary Figure S17). Stratification by MI type indicated that the chronic subgroup had the lowest heterogeneity (*I*^2^ = 27.7%) (Supplementary Figure S18). Baseline CT subgroup analysis confirmed significant NT-proBNP reductions across SCT, IVA, and EECP, but only a non-significant trend for ARNI (Supplementary Figure S19).

Based on the SUCRA analysis, combination therapy with GXST was the most effective intervention for improving NT-proBNP levels in patients (SUCRA 90.1%), followed by QLQX (SUCRA 72.4%) and XT (SUCRA 52.5%).

### 3.11 6 MWT

17 RCTs (19, 23–25, 31, 32, 40, 41, 43, 44, 49, 50, 52, 55–57, 59) reported the 6 MWT, involving 7 CPMs. The results of the network meta-analysis indicated that GXST [MD = 63.84, 95% CI (38.66, 89.02)], QLQX [MD = 48.87, 95% CI (38.96, 58.79)], QSYQ [MD = 37.53, 95% CI (5.42, 69.64)], SXTX [MD = 29.07, 95% CI (3.53, 54.61)], and XT [MD = 29.35, 95% CI (10.95, 47.76)] combined with CT were superior to CT in improving the 6 MWT (Supplementary Table S8).

Adjunctive CPMs significantly extended the 6 MWT distance compared to CT alone [MD = 43.51, 95% CI (37.14, 49.88), *P* < 0.001, *I*^2^ = 63.9%]. After removing 2 studies (23, 39), heterogeneity decreased (*I*^2^ = 46%, *P* < 0.001). Stratification by CPMs category showed that the QLQX subgroup had the largest effect size [MD = 48.03, 95% CI (40.20, 55.86), *I*^2^ = 51.5%] (Supplementary Figure S20). The acute subgroup exhibited the lowest heterogeneity [MD = 44.75, 95% CI (39.20, 50.30), *I*^2^ = 25.8%] (Supplementary Figure S21). Significant 6 MWT benefits were observed across all treatment durations, with the 14-day subgroup exhibiting minimal heterogeneity [MD = 39.50, 95% CI (28.44, 50.56), *I*^2^ = 39.6%; Supplementary Figure S22]. Additionally, subgroup analysis by baseline CT confirmed a consistent and significant extension of 6 MWT distance across all modalities (Supplementary Figure S23).

Based on the SUCRA analysis, combination therapy with GXST was the most effective intervention for improving patients’ 6 min walking distance, with a SUCRA value of 94.4%, followed by QLQX (78.8%) and QSYQ (57.1%).

### 3.12 Adverse events

We systematically extracted adverse event (AE) data from all included RCTs following CONSORT Harms 2022 guidelines. Of 42 studies, 18 RCTs (18–20, 23, 27, 31, 36, 38, 39, 41–44, 46, 50, 51, 56, 58) reported AEs, with comparable incidence rates between experimental (2.65%) and control (2.68%) groups (*P* > 0.05). 9 studies documented no AEs, the remainder reported predominantly mild events typically resolving with symptomatic management (Table 2 and Supplementary Table S9). XT reported a wider variety of AEs, including headache or dizziness, arrhythmia, thirst, frequent urination, nausea and vomiting, hypotension, and gingival bleeding, the latter suggesting the need for attention to its potential effects on coagulation function. QLQX exhibited a higher frequency of specific AEs such as hyperkalemia and limb edema, potentially attributable to the multi-component nature and cardiovascular activity of this herbal preparation. However, AE reporting quality was suboptimal, no study met all CONSORT-Harms criteria (Table 3), with major deficiencies in event definitions, collection procedures, timing, severity grading, and attribution. Most studies focused on short-term safety only, with inadequate renal function monitoring, particularly concerning for cardiorenal assessment in HF.

**Table 2:** Distribution of adverse event types by intervention.

Treatment	Headache or dizziness (n)	Arrhythmia (n)	Thirst (n)	Frequent urination (n)	Nausea or vomiting (n)	Hypotension (n)	Gingival bleeding (n)	Gastrointestinal reaction (n)	Limb edema (n)	Hyperkalemia (n)	Hypersensitivity (n)	Cold Extremities (n)
FFDS	1	1	1	2	–	–	–	–	–	–	–	–
YXS	–	–	–	–	–	–	–	–	–	–	–	–
GXST	2	2	–	–	–	–	–	3	–	–	–	2
QSYQ	–	–	–	–	–	–	–	–	–	–	–	–
SXBX	–	–	–	–	–	–	–	–	–	–	–	–
XNST	–	–	–	–	–	–	–	–	–	–	–	–
XT	4	1	–	–	2	2	1	2	–	–	–	–
QLQX	4	2	–	–	2	2	–	–	2	4	3	–

**Table 3:** Results of CONSORT-harms assessment.

CONSORT—Harms List	Yes/article (proportion/%)	Part is/article (proportion/%)	No/article (proportion/%)
1. If the study collected data on harms and benefits, the title or abstract should so state.	0 (0.0%)	36 (85.7%)	6 (14.3%)
2. If the trial addresses both harms and benefits, the introduction should so state	0 (0.0%)	12 (28.6%)	30 (71.4%)
3. List addressed adverse events with definitions for each (with attention, when relevant, to grading, expected vs. unexpected events, reference to standardized and validated definitions, and description of new definitions)	0 (0.0%)	10 (23.8%)	32 (76.2%)
4. Clarify how harms-related information was collected (mode of data collection, timing, attribution methods, intensity of ascertainment, and harms-related monitoring and stopping rules, if pertinent)	0 (0.0%)	22 (52.4%)	20 (47.6%)
5. Describe plans for presenting and analyzing information on harms (including coding, handling of recurrent events, specification of timing issues, handling of continuous measures, and any statistical analyses)	0 (0.0%)	38 (90.5%)	4 (9.5%)
6. Describe for each arm the participant withdrawals that are due to harms and their experiences with the allocated treatment	0 (0.0%)	5 (11.9%)	37 (88.1%)
7. Provide the denominators for analyses on harms	0 (0.0%)	42 (100%)	0 (0.0%)
8. Present the absolute risk per arm and per adverse event type, grade, and seriousness, and present appropriate metrics for recurrent events, continuous variables, and scale variables, whenever pertinent	0 (0.0%)	23 (54.8%)	19 (45.2%)
9. Describe any subgroup analyses and exploratory analyses for harms	0 (0.0%)	0 (0.0%)	42 (100%)
10. Provide a balanced discussion of benefits and harms with emphasis on study limitations, generalizability, and other sources of information on harms	0 (0.0%)	14(33.3%)	28(66.7%)

### 3.13 Publication bias

**Figure 6:**
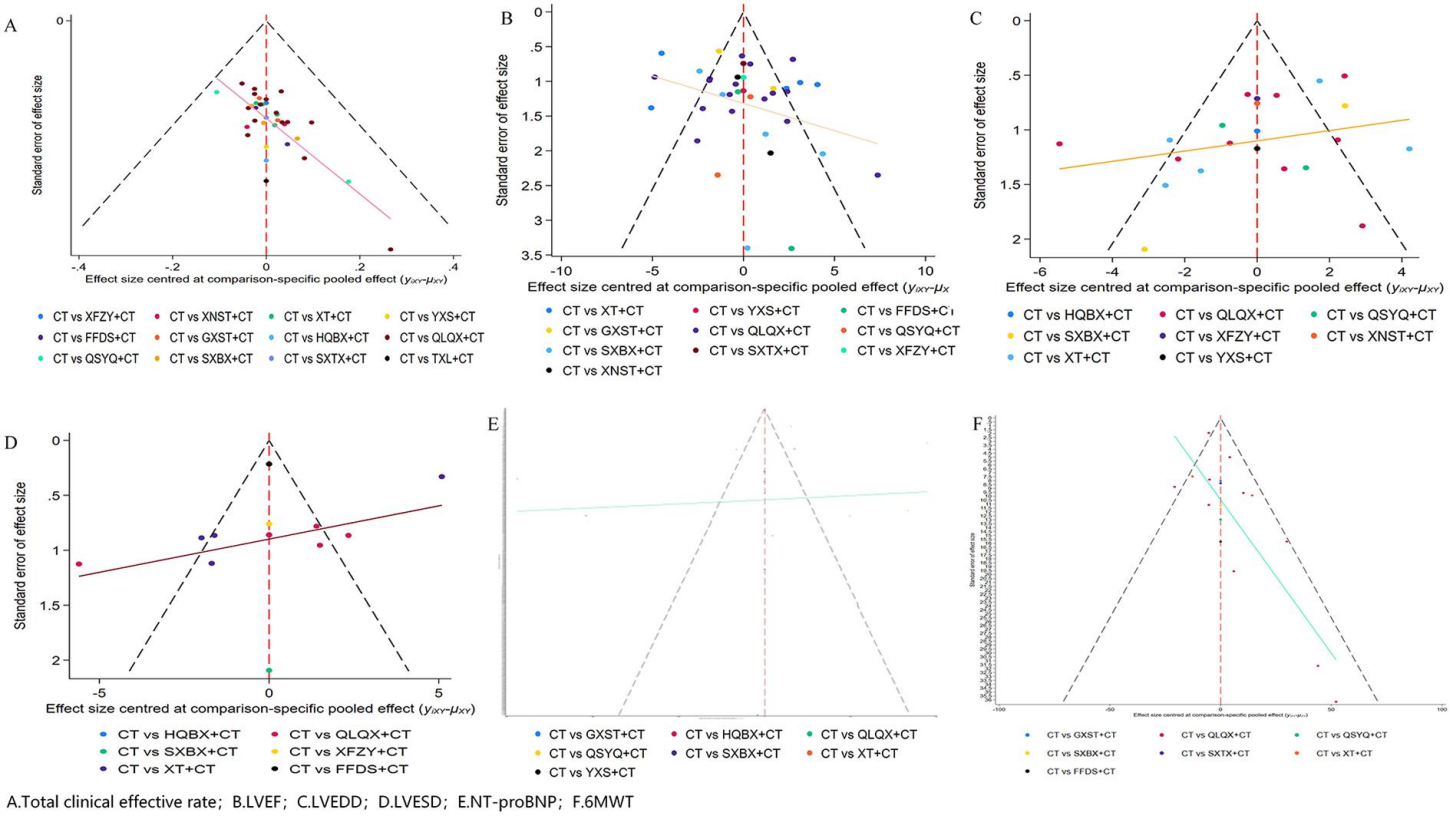
Comparison-adjusted funnel plots for publication bias assessment. Note: **(A)** Total clinical effective rate, **(B)** LVEF, **(C)** LVEDD, **(D)** LVESD, **(E)** NT-proBNP, **(F)** 6MWT.

To comprehensively assess the potential impact of publication bias on the study results, we systematically examined six key outcome indicators using both visual appraisal via comparison-adjusted funnel plots and quantitative statistical tests (Begg's and Egger's tests). Visually, the overall shape of the funnel plots exhibited a certain degree of asymmetry, with some points deviating from the central distribution area (Figure 6). Quantitative assessment using Egger's test verified the visual findings, confirming the presence of statistically significant publication bias or small-study effects for the total clinical effective rate (*P* < 0.001), LVEDD (*P* = 0.043), and 6 MWT (*P* = 0.034). However, no significant publication bias was detected for LVEF (*P* = 0.099), LVESD (*P* = 0.404), and NT-proBNP (*P* = 0.439). These characteristics indicate an undeniable risk of publication bias for certain clinical endpoints, underscoring the need for careful interpretation of the pooled efficacy.

## 4 Discussion

This network meta-analysis of 42 RCTs systematically evaluated the efficacy and safety of 12 oral CPMs combined with CT for treating MI-HF. The findings confirm that adjunctive CPMs significantly improve cardiac function, reverse ventricular remodeling, and enhance exercise tolerance. However, the comparable adverse event rates should be interpreted cautiously; due to poor CONSORT-Harms adherence, these data primarily reflect short-term tolerability rather than definitive safety.

Notably, distinct clinical advantages emerged among the evaluated CPMs: YXS ranked highest for the total clinical effective rate, XT was optimal for improving LVEF and reducing LVESD, SXBX excelled in decreasing LVEDD, and GXST were most effective in lowering NT-proBNP and increasing the 6 MWT distance. Furthermore, QLQX demonstrated robust, comprehensive benefits across multiple key endpoints. The observed differences in efficacy among these formulations align with their distinct multi-target pharmacological mechanisms, providing theoretical support for their differentiated advantages and offering valuable evidence to guide individualized, targeted integrative therapies for MI-HF. From the perspective of TCM theory, the therapeutic rationale for these CPMs is highly consistent with the core pathogenesis of MI-HF. Although most included RCTs did not restrict enrollment to specific TCM phenotypes, the consensus across the literature defines MI-HF as a condition of deficiency in origin and excess in superficiality, with Qi stagnation and blood stasis being the most predominant syndrome. The top-performing formulations identified in our study, such as YXS, QLQX, and XT, are fundamentally designed to supplement Qi, activate blood circulation, and resolve stasis, which perfectly matches this core TCM pathogenesis and explains their broad clinical efficacy.

Mechanistically, the differentiated top-ranking efficacy of these specific formulations perfectly aligns with their distinct, targeted pharmacological pathways identified in modern basic research. Specifically, XT demonstrated superior efficacy in improving systolic geometry and function. Basic studies reveal that it strictly targets mitochondrial structural integrity by upregulating the Pink1, Parkin, and P62 pathways to inhibit excessive mitochondrial autophagy in late-stage HF (60). This energetic and structural repair of cardiomyocytes directly supports its prominent clinical advantage in recovering myocardial contractility. For reducing left ventricular dilation, SXBX exhibited the highest probability of being the optimal intervention. This macroscopic anti-remodeling effect corresponds to its profound microvascular modulation; it mediates GDF15-TRPV4-induced angiogenesis and effectively limits the excessive proliferation and migration of smooth muscle cells via PI3K/AKT/mTOR pathways (61–63). Remarkably, GXST ranked first in both alleviating wall stress and enhancing exercise capacity. This dual clinical distinctiveness is well-explained by its potent vasodilatory properties: it enhances SOD and NOS activities, thereby sharply increasing systemic NO levels (64). The resulting vasodilation not only reduces hemodynamic afterload but also improves peripheral skeletal muscle perfusion, fundamentally boosting exercise tolerance. As a comprehensive formulation, QLQX consistently performed well across almost all functional and structural outcomes. This confirms its established multi-target mechanisms, which include modulating the PI3K/Akt and miR133a-ER stress pathways, mitigating mitochondria-dependent apoptosis, and precisely regulating collagen homeostasis to resolve myocardial fibrosis (65–69). Finally, YXS achieved the highest ranking in the comprehensive overall clinical effective rate. This likely stems from its broad systemic modulation, wherein it activates AMPK/PGC1*α*/GLUT4 to foster robust oxygen delivery and glucose utilization (70), translating cellular energetic stabilization directly into global symptomatic relief. Other formulations like TXL and QSYQ similarly enhance endothelial function and reduce I/R injury via AMPK/mTOR and NLRP3 pathways (71, 72), providing supportive, yet perhaps less specifically targeted, synergistic benefits.

Beyond CPMs, other emerging complementary therapies, such as oxygen-ozone treatment, demonstrate cardioprotective potential in ischemic conditions by modulating endothelial function and oxidative stress (73). Exploring these novel adjunctive modalities could further optimize the multidisciplinary management of MI-HF.

## 5 Limitations

Several limitations of this study should be acknowledged. First, the methodological quality of the included trials was suboptimal due to inadequate reporting of blinding and allocation concealment. Second, baseline therapies in the original studies lacked modern standard treatments, specifically SGLT-2 inhibitors and sGC stimulators, restricting the applicability of the findings to current clinical practice. Third, the lack of prospective Traditional Chinese Medicine syndrome differentiation hinders the precise application of these therapies. Only 4 of the 42 studies stratified patients accordingly, although broader theoretical consensus aligns these therapies with addressing underlying Qi deficiency and blood stasis. Fourth, publication bias and small-study effects were detected for several outcomes. Because exaggerated effect sizes from small trials can propagate through the network and skew probabilistic outcomes, SUCRA rankings must be interpreted cautiously as relative references rather than absolute proofs of clinical superiority (74). Fifth, adverse event reporting was generally incomplete and failed to comply with CONSORT-Harms standards, preventing a reliable evaluation of comprehensive long-term safety.

## 6 Conclusion

Oral CPMs effectively complement conventional therapy for MI-HF with favorable short-term tolerability. Yixinshu Capsules, Xintong Oral Liquid, Shexiang Baoxin Pills, and Guanxinshutong Capsules exhibit distinct targeted advantages, whereas Qili Qiangxin capsules detail comprehensive multi-indicator benefits. However, suboptimal methodological quality, the absence of head-to-head comparisons, and inadequate safety reporting preclude definitive conclusions on absolute rankings and long-term safety. These findings require cautious interpretation and warrant validation through rigorous, large-scale RCTs with extended follow-up.

## Data Availability

The original contributions presented in the study are included in the article/Supplementary Material, further inquiries can be directed to the corresponding author/s.
