# Supplementary material for: The effectiveness and safety of oral Chinese patent medicines in treating myocardial infarction complicated with heart failure: a network meta-analysis of 12 interventions
Source: Front Cardiovasc Med. 2026 May 21;13:1832017. doi: 10.3389/fcvm.2026.1832017 (PMC13233676; doi:10.3389/fcvm.2026.1832017)
Supplement: Supplementary file 1 [file Datasheet1.pdf]

Supplementary Materials

Supplementary Tables

Supplementary Table S1 Basic information of all the papers

Supplementary Table S2 Details of Chinese patent medicines included in this study

Supplementary Table S3 Results of network Meta-Analysis(Total Clinical Effective Rate)

Supplementary Table S4 Results of network Meta-Analysis (LVEF)

Supplementary Table S5 Results of network Meta-Analysis (LVEDD)

Supplementary Table S6 Results of network Meta-Analysis (LVESD)

Supplementary Table S7 Results of network Meta-Analysis (NT-proBNP)

Supplementary Table S8 Results of network Meta-Analysis (6MWT)

Supplementary Table S9 Occurrence of Adverse Events in Included Studies

Supplementary Figures

Supplementary Figure S1. Forest plot demonstrating the overall clinical effective rate of oral Chinese patent medicines (CPMs) combined with conventional treatment (CT).

Supplementary Figure S2. Surface under the cumulative ranking curve (SUCRA) plots for the total clinical effective rate.

Supplementary Figure S3. SUCRA plots for LVEF.

Supplementary Figure S4. SUCRA plots for LVEDD.

Supplementary Figure S5. SUCRA plots for LVESD.

Supplementary Figure S6. SUCRA plots for NT-proBNP.

Supplementary Figure S7. SUCRA plots for 6MWT.

Supplementary Figure S8. Forest plot of the overall effect of oral CPMs combined with conventional treatment on LVEF.

Supplementary Figure S9. Subgroup analysis of LVEF based on different CT.

Supplementary Figure S10. Subgroup analysis of LVEF structured by the duration of combination therapy.

Supplementary Figure S11. Subgroup analysis of LVEF stratified by the stage of myocardial infarction(MI).

Supplementary Figure S12. Subgroup analysis of LVEF categorized by specific CPMs.

Supplementary Figure S13. Subgroup analysis of LVEDD categorized by specific CPMs.

Supplementary Figure S14. Subgroup analysis of LVEDD based on different CT background regimens.

Supplementary Figure S15. Subgroup analysis of LVESD based on different CT background regimens.

Supplementary Figure S16. Subgroup analysis of LVESD categorized by specific CPMs.

Supplementary Figure S17. Subgroup analysis of NT-proBNP categorized by specific CPMs.

Supplementary Figure S18. Subgroup analysis of NT-proBNP stratified by the stage of MI.

Supplementary Figure S19. Subgroup analysis of NT-proBNP based on different CT background regimens.

Supplementary Figure S20. Subgroup analysis of the 6MWT distance categorized by specific CPMs.

Supplementary Figure S21. Subgroup analysis of the 6MWT distance stratified by the stage of MI.

Supplementary Figure S22. Subgroup analysis of the 6MWT distance structured by the duration of combination therapy.

Supplementary Figure S23. Subgroup analysis of the 6MWT distance based on different CT background regimens.

Appendix S1 Search strategy

Supplementary Table S1 Basic information of all the papers

Paper	Sample size Total(Treatment group/Control group)	Gender (M/F) Treatment group	Control group	Age(years) Treatment group	Control group	Intervention Treatment group	Intervention Control group	Duration of treatment	Outcomes	Disease course
Huang ZX,2022[18]	60 (30/30)	12/18	20/10	58.41±4.16	59.62±4.51	FFDS+CT	SCT	180d	①②⑤	A
Ge YH,2015[19]	66 (33/33)	19/14	17/16	63±10	61±11	YXS+CT	SCT	90d	①②④⑥	C
Zhang ZL,2020[20]	94 (47/47)	33/14	30/17	60.4±6.6	61.3±7.1	FFDS+CT+TPT	SCT	7d	①②	A
Zhang K,2021[21]	124 (62/62)	33/29	35/27	55±3.4	54.8±2.3	XFZY+CT	SCT	30d	①②③④	A
Li F,2019[22]	61 (30/31)	18/13	17/13	57±5	56.8±5.2	GXST+CT+CLP	SCT	180d	①②	A
Shi H,2022[23]	124 (62/62)	43/19	41/21	62.69±4.8	62.63±4.71	GXST+CT+CLP	SCT	3d	①②⑤⑥	A
Li YQ,2019[24]	66 (33/33)	18/15	17/16	64.2±5.37	64.12±5.34	HQBX+CT	SCT	14d	①③④⑥	A
Dou MM,2023[25]	90 (45/45)	28/17	30/15	63.9±8.7	64.2±8.3	QSYQ+CT+NKL	SCT	30d	②③⑥	A
Zhang Li,2024[26]	48 (24/24)	15/9	16/8	63.47±5.31	61.69±5.78	QSYQ+CT+SKB	ARNI	60d	①③	C
Ren LF,2017[27]	100 (42/58)	/	/	/	/	QSYQ+CT	SCT	30d	①②⑤	C
Qiu CY,2019[28]	101 (50/51)	29/21	26/24	61.29±6.36	62.08±6.78	SXTX+CT	SCT	90d	①②	C
Liao R,2024[29]	43 (21/22)	12/10	11/10	63.97±6.89	63.62±7.01	SXBX+CT	SCT	90d	②③	U
Guo XL,2012[30]	92 (34/58)	23/35	14/20	68.2±9.33	67.8±10.72	SXBX+CT	SCT	14d	①②	C
Li MC,2017[31]	114 (57/57)	30/27	32/25	63.5±10.5	62.8±10.7	SXBX+CT+EECP	EECP	60d	①②③⑥	A
Wei FY,2024[32]	80 (40/40)	29/11	25/15	68.58±8.57	66.88±8.9	SXBX+CT	ARNI	30d	①②⑤⑥	U
Kong FY,2015[33]	60 (30/30)	14/16	13/17	68±10	70±13	SXBX+CT+EECP	EECP	60d	②⑤	A
Liu XL,2015[34]	60 (30/30)	18/12	16/14	62.5±5.2	61.5±4.7	SXBX+CT	SCT	14d	②③④	A
Shi Q,2006[35]	60 (30/30)	20/10	17/13	61.8±6.3	62.3±7.2	TXL+CT	SCT	90d	①③	A
Li WJ,2019[36]	96 (48/48)	29/19	27/21	55.78±3.72	57.11±3.83	XNST+CT+BSL	SCT	180d	①②	U
Shi Z,2013[37]	82 (41/41)	/	/	/	/	XNST+CT	SCT	12m	①②③	C
He Q,2022[38]	108 (54/54)	38/16	35/19	61.25±6.78	58.45±6.31	XT+CT+ENL	SCT	8d	①②③④	A
Shi XM,2024[39]	86 (43/43)	22/21	21/22	65.29±3.31	64.17±3.14	XT+CT+rhBNP	IVA	14d	①②③④⑤	A
Xu PX,2024[40]	86 (43/43)	25/18	23/20	55.59±4.38	61.98±3.38	XT+CT+rhBNP	IVA	90d	①②③④⑥	A

Paper	Sample size Total(Treatment group/Control group)	Gender (M/F) Treatment group	Control group	Age(years) Treatment group	Control group	Intervention Treatment group	Intervention Control group	Duration of treatment	Outcomes	Disease course
Chen QJ,2020[41]	100 (50/50)	34/16	32/18	63.72±6.85	62.53±5.61	XT+CT+XT	SCT	30d	②③⑤⑥	A
Yan HY,2020[42]	126 (63/63)	38/25	41/22	62.21±5.28	62.05±4.34	XT+CT+rhBNP	IVA	30d	①②③④	A
Chen WJ,2021[43]	108 (54/54)	30/24	30/24	59.67±6.48	59.91±7.11	QLQX+CT+MLN	IVA	90d	①②③⑥	A
Zhu WY,2015[44]	60 (29/31)	/	/	/	/	QLQX+CT	SCT	14d	②⑤⑥	A
Pang J,2014[45]	107 (54/53)	29/24	30/24	/	/	QLQX+CT	SCT	90d	①②③	A
Xu X,2024[46]	82 (41/41)	20/21	19/22	60.27±9.12	59.35±8.64	QLQX+CT+rhBNP	IVA	21d	①②③④	A
Luo F,2019[47]	60 (30/30)	16/15	15/14	56.97±8.82	57.14±8.06	QLQX+CT	SCT	90d	①②③④	C
Xi K,2011[48]	60 (30/30)	/	/	/	/	QLQX+CT	SCT	30d	①⑤	U
Han L,2024[49]	92 (46/46)	28/18	26/20	68.45±3.42	68.34±3.31	QLQX+CT+SKB	ARNI	90d	①②③④⑤⑥	A
Xie ZQ,2022[50]	100 (50/50)	32/18	30/20	71.14±2.08	71.25±2.02	QLQX+CT+SKB	ARNI	90d	①②⑤⑥	A
Pan YK,2024[51]	110 (55/55)	30/25	28/27	53.2±5.7	53.5±5.49	QLQX+CT+LXC	IVA	60d	①②	A
Dai SR,2022[52]	90 (45/45)	26/19	27/18	70.13±5.48	69.98±5.27	QLQX+CT+rhBNP	IVA	14d	②⑤⑥	A
Bai LQ,2013[53]	40 (20/20)	12/8	11/9	52±4.2	45±3.7	QLQX+CT	SCT	3m	①②⑤	A
Qiao DF,2018[54]	122 (61/61)	34/27	33/28	54.5±2.2	54.3±2.7	QLQX+CT	SCT	90d	①②③④	C
Chen YQ,2020[55]	102 (51/51)	33/18	31/20	63.16±11.57	62.85±11.73	QLQX+CT	SCT	180d	①②③⑤⑥	C
Song JY,2020[56]	167 (84/83)	45/38	48/36	63.12±7.41	62.25±7.65	QLQX+CT+MLN	IVA	90d	①②③⑤⑥	A
Liu Y,2023[57]	82 (41/41)	24/17	21/20	71.58±8.46	70.34±9.15	QLQX+CT+LXC	IVA	21d	①②⑥	A
Fu JZ,2010[58]	64 (32/32)	20/12	19/13	60.38±10.6	61.3±10.1	QLQX+CT	SCT	21d	①②	A
Zhang N,2022[59]	102 (45/57)	23/34	29/16	66.2±2.9	65.3±3.5	QLQX+CT+rhBNP	IVA	14d	①②③④⑤⑥	A

Note: Disease course: A, acute phase; C, chronic phase; U, unclear. d: day. **Interventions:** CT, conventional Western medicine; FFDS, Compound Danshen Dripping Pills; YSS, Yixinshu Capsules; TXL, Tongxinluo Capsules; XFZY, Xuefu Zhuyu Capsules; GXST, Guanxinshutong Capsules; XNST, Xinnaoshutong Capsules; QLQX, Qili Qiangxin Capsules; SXTX, Shexiang Tongxin Dripping Pills; SXBX, Shexiang Baoxin Pills; HQBX, Huangqi Baoxin Granules; XT, Xintong Oral Liquid; XTM, simulated Xintong Oral Liquid (placebo); QSYQ, Qishen Yiqi Dripping Pills. SCT (Standard Conventional Treatment): Classic guideline-directed medical therapy primarily comprising oral antiplatelet agents, ACEI/ARB, beta-blockers, mineralocorticoid receptor antagonists, statins, and diuretics, alongside routine individualized adjuncts. ARNI: Enhanced therapy incorporating Angiotensin Receptor-Neprilysin Inhibitor (Sacubitril/Valsartan). IVA (Intravenous Vasoactive Agents): Intensive therapies typically required during acute decompensation (e.g., intravenous rhBNP, Levosimendan, or Milrinone). EECP (Enhanced External Counterpulsation): Device-assisted therapy combined with standard conventional treatment. **Outcomes:** ① Total clinical effective rate; ② LVEF; ③ LVEDD; ④ LVESD; ⑤NT-proBNP ; ⑥ 6MWT.

Supplementary Table S2 Details of Chinese patent medicines included in this study

Chinese Patent Medicine	Manufacturer	Dosage	Ingredients
Qili Qiangxin Capsules	Shijiazhuang Yiling Pharmaceutical Co., Ltd.<br>National Medical Product Approval No. Z20040141	4 capsules tid	Astragalus membranaceus, Ginseng, Processed Aconite Root, Salvia miltiorrhiza, Descurainiae Semen, Alisma Rhizome, Polygonatum Odoratum, Cinnamon Twig, Safflower, Chinese Silkvine Root-bark, Dried Tangerine Peel
Compound Danshen Dripping Pills	Tianjin Tasly Pharmaceutical Co., Ltd.<br>National Medical Product Approval No. Z10950111	10 pills tid	Salvia miltiorrhiza, Panax notoginseng, Borneol
Shexiang Baoxin Pills	Shanghai Hutchison Pharmaceuticals Ltd.<br>National Medical Product Approval No. Z31020068	2 pills tid	Artificial Musk, Ginseng Extract, Artificial Bezoar, Cinnamon Bark, Styrax, Toad Venom, Borneol
Yixinshu Capsules	Guizhou Bailing Group Pharmaceutical Co., Ltd.	4 capsules tid	Ginseng, Astragalus membranaceus, Salvia miltiorrhiza, Ophiopogon Japonicus, Schisandra Fruit, Szechwan Lovage Rhizome, Hawthorn Fruit
Xuefu Zhuyu Capsules	Tianjin Hongrentang Pharmaceutical Co., Ltd.<br>National Medical Product Approval No. Z12020223	6 capsules bid	Peach Kernel, Safflower, Angelica Sinensis, Szechwan Lovage Rhizome, Rehmannia Root, Red Peony Root, Achyranthes Root, Bupleurum Root, Bitter Orange Peel, Platycodon Root, Licorice Root
Xintong Oral Liquid	Lunan Houpu Pharmaceutical Co., Ltd.<br>National Medical Product Approval No. Z10920014	10 mL tid	Astragalus membranaceus, Codonopsis, Ophiopogon Japonicus, Polygonum Multiflorum, Epimedium, Wild Chrysanthemum Flower, Angelica Sinensis, Salvia miltiorrhiza, Chinese Honeylocust Thorn, Sargassum, Kelp, Oyster Shell, Immature Bitter Orange
Xinnaoshutong Capsules	Jilin Aodong Taonan Pharmaceutical Co., Ltd.<br>National Medical Product Approval No. Z22021965	2 capsules tid	Tribulus terrestris
Tongxinluo Capsules	Shijiazhuang Yiling Pharmaceutical Co., Ltd.<br>National Medical Product Approval No. Z19980015	4 capsules tid	Red Peony Root, Salvia miltiorrhiza, Ground Beetle, Centipede, Scorpion, Cicada Slough, Rosewood, Borneol, Sandalwood, Frankincense, Sour Jujube Seed
Shexiang Tongxin Dripping Pills	Inner Mongolia Conba Pharmaceutical Co., Ltd. Shenglong Branch<br>National Medical Product Approval No. Z20080018	2 pills tid	Artificial Musk, Total Ginsenosides of Ginseng Stems and Leaves, Toad Venom, Salvia miltiorrhiza, Artificial Bezoar, Bear Bile Powder, Borneol
Qishen Yiqi Dripping Pills	Tasly Pharmaceutical Group Co., Ltd.<br>National Medical Product Approval No. Z20030139	1 sachet tid	Astragalus membranaceus, Salvia miltiorrhiza, Panax notoginseng, Rosewood
Huangqi Baoxin Granules	Sichuan Bailee Pharmaceutical Co., Ltd.<br>National Medical Product Approval No. Z19993381	1 sachet tid	Astragalus membranaceus, Salvia miltiorrhiza, Angelica Sinensis, Szechwan Lovage Rhizome, Hawthorn Fruit, Licorice Root
Guanxinshutong Capsules	Shaanxi Buchang Pharmaceutical Co., Ltd.<br>National Medical Product Approval No. Z20020055	3 capsules tid	Nepali Hog Plum, Salvia miltiorrhiza, Clove, Borneol, Tabasheer

Supplementary Table S3 Results of network Meta-Analysis(Total Clinical Effective Rate)

Intervention	RR[95%CI]												
	**YXS+CT**	**TXL+CT**	**SXBX+CT**	**XNST+CT**	**FFDS+CT**	**HQBX+CT**	**XT+CT**	**QLQX+CT**	**GXST+CT**	**XFZY+CT**	**SXTX+CT**	**QSYS+CT**	**CT**
YXS+CT	1.00												
TXL+CT	0.98 (0.67,1.45)	1.00											
SXBX+CT	1.07 (0.82,1.40)	1.09 (0.79,1.51)	1.00										
XNST+CT	1.08 (0.82,1.43)	1.09 (0.78,1.53)	1.01 (0.84,1.20)	1.00									
FFDS+CT	1.11 (0.84,1.46)	1.13 (0.80,1.57)	1.03 (0.87,1.23)	1.03 (0.84,1.25)	1.00								
HQBX+CT	1.11 (0.77,1.59)	1.12 (0.75,1.69)	1.03 (0.77,1.38)	1.03 (0.76,1.39)	1.00 (0.74,1.35)	1.00							
XT+CT	1.13 (0.87,1.45)	1.14 (0.83,1.57)	1.05 (0.91,1.21)	1.04 (0.89,1.23)	1.02 (0.87,1.19)	1.02 (0.77,1.35)	1.00						
QLQX+CT	1.14 (0.89,1.45)	1.15 (0.85,1.57)	1.06 (0.94,1.19)	1.06 (0.91,1.22)	1.03 (0.89,1.18)	1.03 (0.78,1.35)	1.01 (0.92,1.11)	1.00					
GXST+CT	1.14 (0.87,1.49)	1.16 (0.84,1.61)	1.06 (0.91,1.25)	1.06 (0.88,1.27)	1.03 (0.86,1.23)	1.03 (0.77,1.38)	1.01 (0.88,1.17)	1.00 (0.89,1.14)	1.00				
XFZY+CT	1.16 (0.87,1.54)	1.17 (0.83,1.66)	1.08 (0.89,1.30)	1.07 (0.87,1.32)	1.04 (0.85,1.28)	1.04 (0.77,1.42)	1.03 (0.86,1.23)	1.02 (0.86,1.20)	1.01 (0.83,1.23)	1.00			
SXTX+CT	1.16 (0.86,1.57)	1.18 (0.82,1.69)	1.08 (0.87,1.34)	1.08 (0.85,1.36)	1.05 (0.83,1.32)	1.05 (0.76,1.45)	1.03 (0.84,1.26)	1.02 (0.84,1.23)	1.02 (0.82,1.27)	1.00 (0.79,1.28)	1.00		
QSYS+CT	1.24 (0.95,1.63)	1.26 (0.90,1.75)	1.16 (0.98,1.37)	1.15 (0.95,1.39)	1.12 (0.93,1.35)	1.12 (0.83,1.50)	1.10 (0.95,1.28)	1.09 (0.96,1.24)	1.09 (0.92,1.29)	1.07 (0.88,1.31)	1.07 (0.85,1.34)	1.00	
CT	1.35 (1.06,1.72)	1.37 (1.01,1.86)	1.26 (1.13,1.40)	1.25 (1.09,1.44)	1.22 (1.06,1.39)	1.22 (0.93,1.59)	1.20 (1.10,1.30)	1.18 (1.13,1.24)	1.18 (1.05,1.33)	1.17 (1.00,1.37)	1.16 (0.96,1.40)	1.09 (0.96,1.23)	1.00

Supplementary Table S4 Results of network Meta-Analysis (LVEF)

Intervention	MD[95%CI]										
	XT+CT	QLQX+CT	YXS+CT	FFDS+CT	GXST+CT	XNST+CT	SXBX+CT	XFZY+CT	SXTX+CT	QSYQ+CT	CT
XT+CT	0										
QLQX+CT	2.60[-0.21,5.41]	0									
YXS+CT	2.26[-3.77,8.29]	-0.34[-6.03,5.35]	0								
FFDS+CT	3.61[-1.61,8.83]	1.01[-3.81,5.83]	1.35[-5.85,8.54]	0							
GXST+CT	3.65[-0.84,8.13]	1.04[-2.97,5.06]	1.39[-5.29,8.06]	0.04[-5.91,5.99]	0						
XNST+CT	4.03[-0.76,8.82]	1.42[-2.93,5.78]	1.77[-5.12,8.65]	0.42[-5.77,6.61]	0.38[-5.20,5.96]	0					
SXBX+CT	4.74[1.07,8.41]	2.14[-0.94,5.22]	2.48[-3.68,8.64]	1.13[-4.23,6.50]	1.09[-3.56,5.75]	0.71[-4.24,5.67]	0				
XFZY+CT	5.22[-0.68,11.12]	2.62[-2.93,8.17]	2.96[-4.74,10.66]	1.61[-5.47,8.70]	1.57[-4.99,8.13]	1.19[-5.58,7.97]	0.48[-5.56,6.52]	0			
SXTX+CT	6.23[0.44,12.02]	3.63[-1.81,9.06]	3.97[-3.65,11.59]	2.62[-4.37,9.62]	2.58[-3.88,9.05]	2.20[-4.47,8.88]	1.49[-4.44,7.42]	1.01[-6.50,8.52]	0		
QSYQ+CT	6.18[1.22,11.15]	3.58[-0.97,8.13]	3.92[-3.09,10.93]	2.57[-3.75,8.90]	2.54[-3.20,8.27]	2.16[-3.82,8.13]	1.44[-3.69,6.57]	0.96[-5.94,7.86]	-0.05[-6.85,6.75]	0	
CT	9.26[6.83,11.70]	6.66[5.26,8.06]	7.00[1.48,12.52]	5.65[1.04,10.27]	5.61[1.85,9.38]	5.23[1.11,9.36]	4.52[1.77,7.27]	4.04[-1.33,9.41]	3.03[-2.22,8.28]	3.08[-1.25,7.40]	0

Supplementary Table S5 Results of network Meta-Analysis (LVEDD)

Intervention	MD[95%CI]								
	SXBX+CT	QLQX+CT	XFZY+CT	XNST+CT	YXS+CT	XT+CT	HQBX+CT	QSYQ+CT	CT
SXBX+CT	0								
QLQX+CT	-8.67[-13.02,-4.32]	0							
XFZY+CT	-9.20[-15.61,-2.79]	-0.53[-5.85,4.80]	0						
XNST+CT	-9.22[-15.64,-2.79]	-0.55[-5.90,4.80]	-0.02[-7.14,7.10]	0					
YXS+CT	-9.32[-15.98,-2.66]	-0.65[-6.28,4.98]	-0.12[-7.46,7.22]	-0.10[-7.45,7.25]	0				
XT+CT	-9.54[-14.17,-4.91]	-0.87[-3.84,2.10]	-0.34[-5.90,5.22]	-0.32[-5.91,5.26]	-0.22[-6.07,5.63]	0			
HQBX+CT	-11.39[-17.95,-4.83]	-2.72[-8.23,2.79]	-2.19[-9.43,5.05]	-2.17[-9.43,5.09]	-2.07[-9.54,5.40]	-1.85[-7.59,3.89]	0		
QSYQ+CT	-11.31[-16.80,-5.83]	-2.65[-6.81,1.52]	-2.12[-8.40,4.17]	-2.10[-8.40,4.21]	-2.00[-8.54,4.55]	-1.77[-6.24,2.69]	0.07[-6.36,6.51]	0	
CT	-15.32[-19.29,-11.34]	-6.65[-8.41,-4.89]	-6.12[-11.14,-1.10]	-6.10[-11.15,-1.05]	-6.00[-11.34,-0.66]	-5.78[-8.17,-3.39]	-3.93[-9.15,1.29]	-4.00[-7.78,-0.23]	0

Supplementary Table S6 Results of network Meta-Analysis (LVESD)

Intervention	MD[95%CI]						
	XT+CT	SXBX+CT	QLQX+CT	XFZY+CT	HQBX+CT	FFDS+CT	CT
XT+CT	0						
SXBX+CT	-1.36[-9.53,6.80]	0					
QLQX+CT	-2.14[-6.50,2.22]	-0.78[-8.82,7.26]	0				
XFZY+CT	-3.95[-11.17,3.26]	-2.59[-12.47,7.29]	-1.81[-8.89,5.26]	0			
HQBX+CT	-4.09[-11.35,3.17]	-2.73[-12.65,7.19]	-1.95[-9.07,5.17]	-0.14[-9.29,9.01]	0		
FFDS+CT	-5.98[-13.05,1.09]	-4.62[-14.40,5.16]	-3.84[-10.77,3.09]	-2.03[-11.03,6.97]	-1.89[-10.93,7.15]	0	
CT	-7.26[-10.50,-4.02]	-5.90[-13.39,1.59]	-5.12[-8.04,-2.20]	-3.31[-9.75,3.13]	-3.17[-9.66,3.32]	-1.28[-7.56,5.00]	0

Supplementary Table S7 Results of network Meta-Analysis (NT-proBNP)

Intervention	MD[95%CI]							
	GXST+CT	QLQX+CT	XT+CT	HQBX+CT	SXBX+CT	YXS+CT	QSYQ+CT	CT
GXST+CT	0							
QLQX+CT	-308.49 [-981.01,364.03]	0						
XT+CT	-474.94 [-1247.80,297.93]	-166.45 [-664.61,331.70]	0					
HQBX+CT	-513.92 [-1407.90,380.06]	-205.43 [-876.28,465.41]	-38.98 [-810.39,732.43]	0				
SXBX+CT	-524.52 [-1432.43,383.39]	-216.03 [-905.34,473.28]	-49.58 [-837.10,737.94]	-10.60 [-917.27,896.07]	0			
YXS+CT	-639.82 [-1532.90,253.26]	-331.33 [-1000.98,338.31]	-164.88 [-935.24,605.48]	-125.90 [-1017.71,765.91]	-115.30 [-1021.09,790.49]	0		
QSYQ+CT	-672.57 [-1563.54,218.40]	-364.08 [-1030.91,302.74]	-197.63 [-965.55,570.29]	-158.65 [-1048.35,731.05]	-148.05 [-1051.76,755.66]	-32.75 [-921.55,856.05]	0	
CT	-787.82 [-1420.85,-154.79]	-479.33 [-706.41,-252.26]	-312.88 [-756.27,130.51]	-273.90 [-905.14,357.34]	-263.30 [-914.14,387.54]	-148.00 [-777.97,481.97]	-115.25 [-742.22,511.72]	0

Supplementary Table S8 Results of network Meta-Analysis (6MWT)

Intervention	MD[95%CI]							
	GXST+CT	QLQX+CT	QSYS+CT	FFDS+CT	SXTX+CT	XT+CT	SXBX+CT	CT
GXST+CT	0							
QLQX+CT	14.97[-12.10,42.03]	0						
QSYS+CT	26.31[-14.50,67.12]	11.34[-22.27,44.96]	0					
FFDS+CT	30.76[-13.71,75.23]	15.79[-22.18,53.77]	4.45[-44.28,53.18]	0				
SXTX+CT	34.77[-1.09,70.63]	19.80[-7.59,47.20]	8.46[-32.57,49.49]	4.01[-40.66,48.68]	0			
XT+CT	34.49[3.30,65.67]	19.52[-1.04,40.08]	8.18[-28.84,45.19]	3.73[-37.29,44.74]	-0.28[-31.77,31.20]	0		
SXBX+CT	41.28[2.55,80.01]	26.31[-4.74,57.37]	14.97[-28.59,58.53]	10.52[-36.48,57.52]	6.51[-32.45,45.47]	6.79[-27.91,41.50]	0	
CT	63.84[38.66,89.02]	48.87[38.96,58.79]	37.53[5.42,69.64]	33.08[-3.57,69.73]	29.07[3.53,54.61]	29.35[10.95,47.76]	22.56[-6.86,51.98]	0

Supplementary Table S9 Occurrence of Adverse Events in Included Studies

Study	Intervention	Adverse Events(n)	
		T	C
Huang ZX[16]	FFDS+CT	0Headache, 1Dizziness, 1Palpitation	2Headache, 1Dizziness, 1Palpitation
Ge YH[17]	YXS+CT	-	-
Zhang ZL[18]	FFDS+CT	1Thirst, 2Frequent urination	1Thirst, 1Frequent urination
Shi H[21]	GXST+CT	3Gastrointestinal reaction, 2Headache or dizziness, 2Cold extremities, 1Bradycardia, 1Palpitation	2Gastrointestinal reaction, 2Headache or Dizziness, 1Cold Extremities, 1Bradycardia, 0Palpitation
Ren LF[25]	QSYQ+CT	-	-
Li MC[29]	SXBX+CT	-	-
Li WJ[34]	XNST+CT	-	-
He Q[36]	XT+CT	2Nausea or vomiting, 2Headache, 2Hypotension, 1Gingival Bleeding	1Nausea or vomiting, 1Headache, 1Hypotension, 2Gingival Bleeding
Shi XM[37]	XT+CT	1Arrhythmia, 2Gastrointestinal reaction, 2Headache or dizziness	2Arrhythmia, 2Gastrointestinal reaction, 1Headache or dizziness
Chen QJ[39]	XT+CT	-	-
Yan HY[40]	XT+CT	-	-
Chen WJ[41]	QLQX+CT	-	-
Zhu WY[42]	QLQX+CT	-	3Palpitations
Xu X[44]	QLQX+CT	2Nausea or vomiting, 2Dizziness, 3Hypersensitivity	2Nausea or vomiting, 1Dizziness, 2Hypersensitivity
Xie ZQ[48]	QLQX+CT	2Hypotension, 1Dizziness, 4Hyperkalemia	2Hypotension, 1Dizziness, 3Hyperkalemia
Pan YK[49]	QLQX+CT	2Limb Edema, 1Headache, 2Arrhythmia	4Limb Edema, 2Headache, 8Arrhythmia
Song JY[54]	QLQX+CT	-	-
Fu JZ[56]	QLQX+CT	-	-

Supplementary Figure S1. Forest plot demonstrating the overall clinical effective rate of oral Chinese patent medicines (CPMs) combined with conventional treatment (CT).

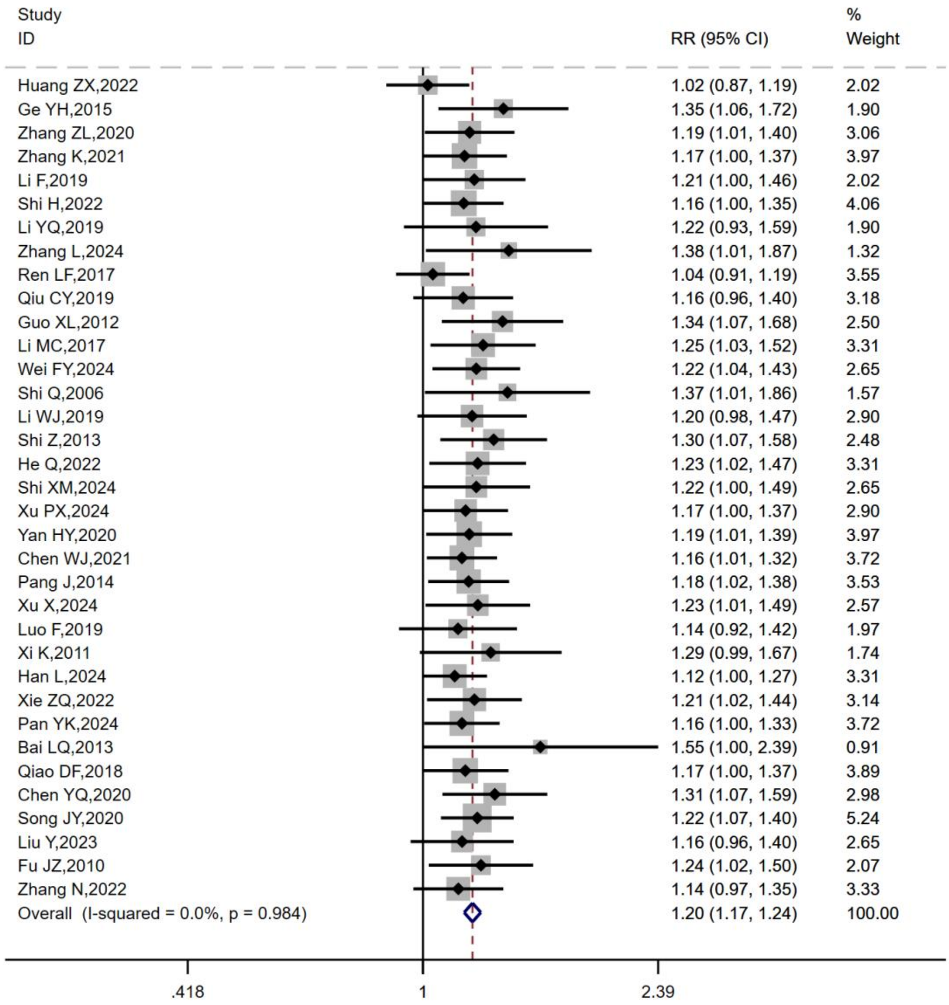

Note: The meta-analysis evaluates the dichotomous outcome of the total clinical effective rate. The solid vertical line at 1.0 represents the line of no effect. Black diamonds with horizontal lines indicate the Risk Ratio (RR) and 95% confidence intervals (CI) for each individual trial. The open diamond at the bottom represents the overall pooled RR, indicating that the combination therapy is significantly superior to conventional treatment alone (RR = 1.20, 95% CI [1.17, 1.24]). Notably, the analysis exhibited an exceptionally high degree of consistency across all included studies, with zero statistical heterogeneity (I^2 = 0.0%, P = 0.984).

Supplementary Figure S2. Surface under the cumulative ranking curve (SUCRA) plots for the total clinical effective rate.

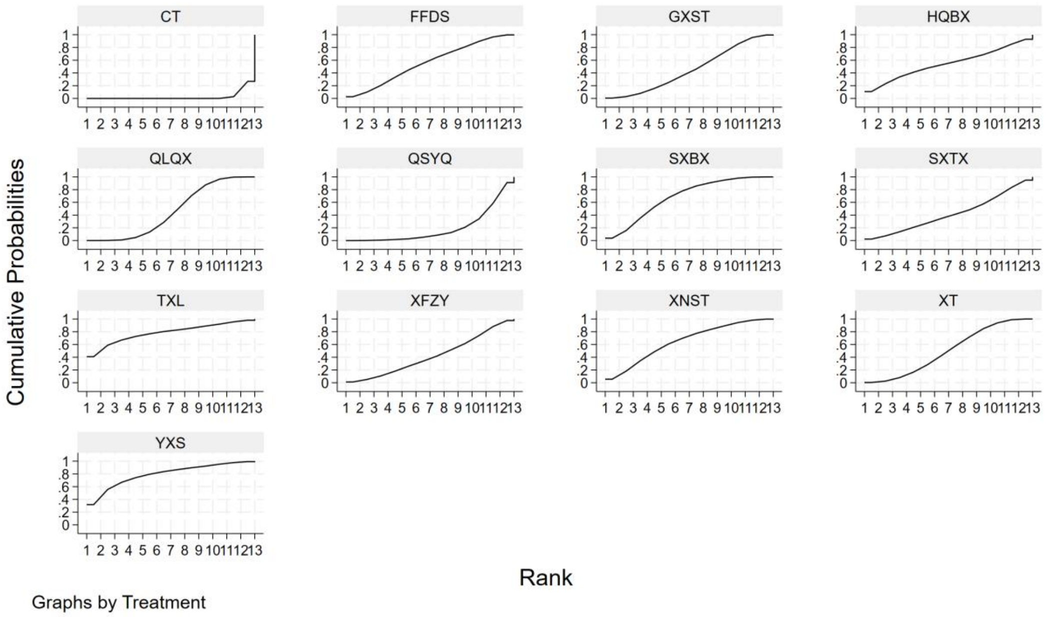

Supplementary Figure S3. SUCRA plots for LVEF.

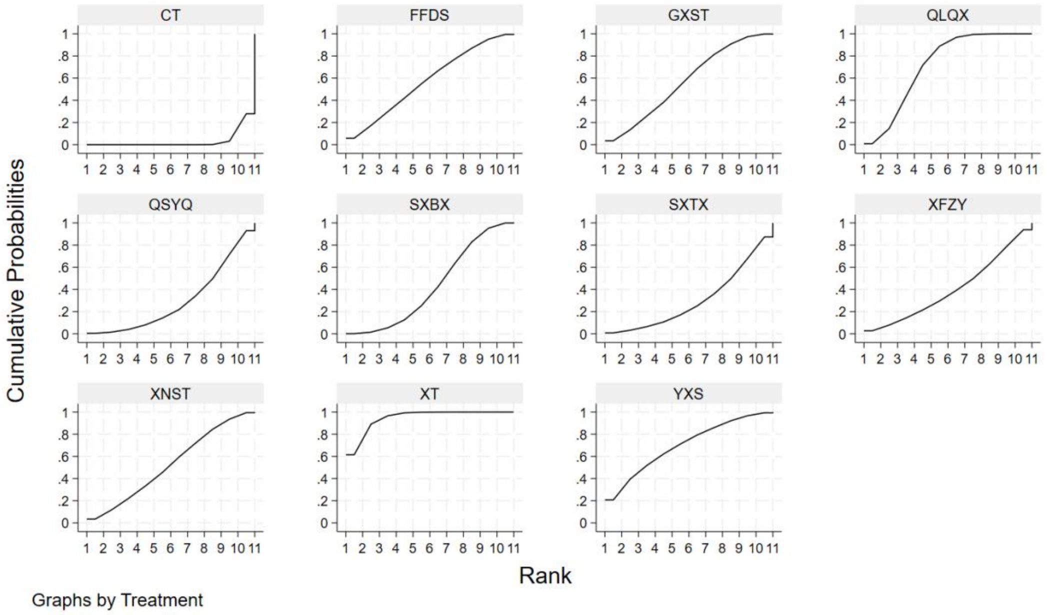

Supplementary Figure S4. SUCRA plots for LVEDD.

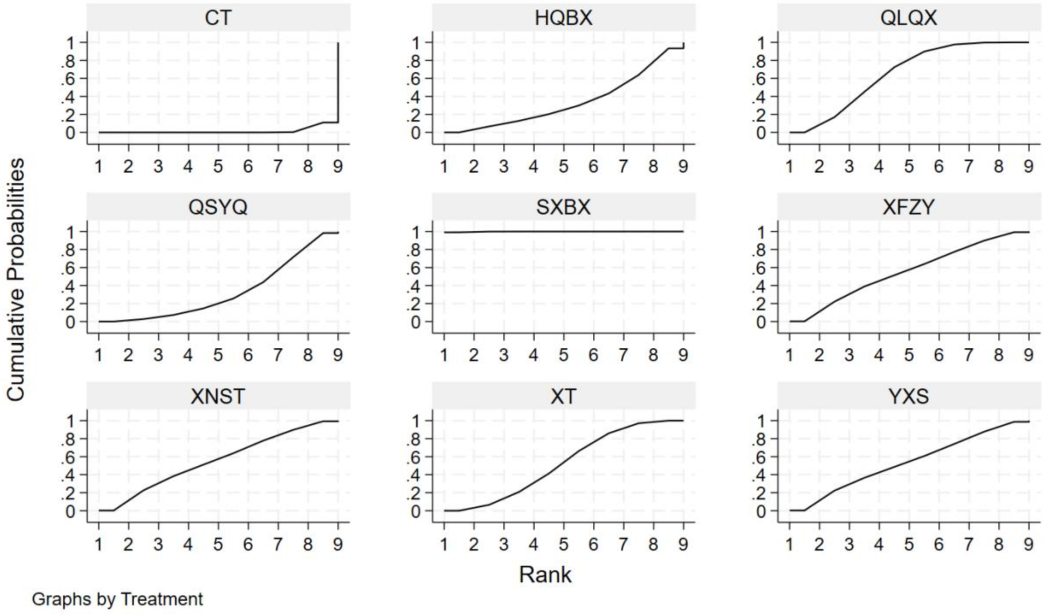

Supplementary Figure S5. SUCRA plots for LVESD.

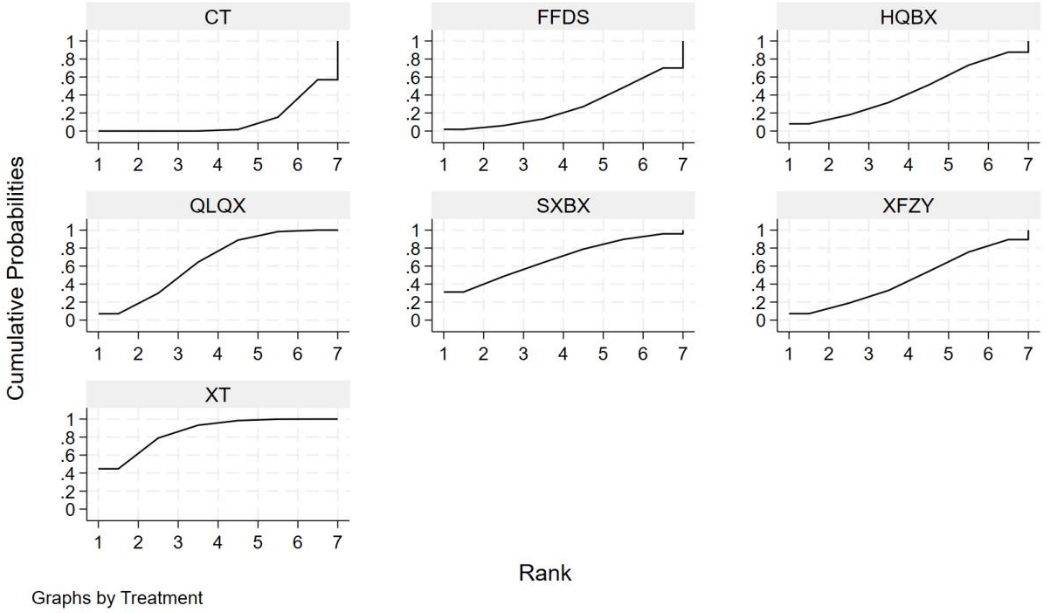

Supplementary Figure S6. SUCRA plots for NT-proBNP.

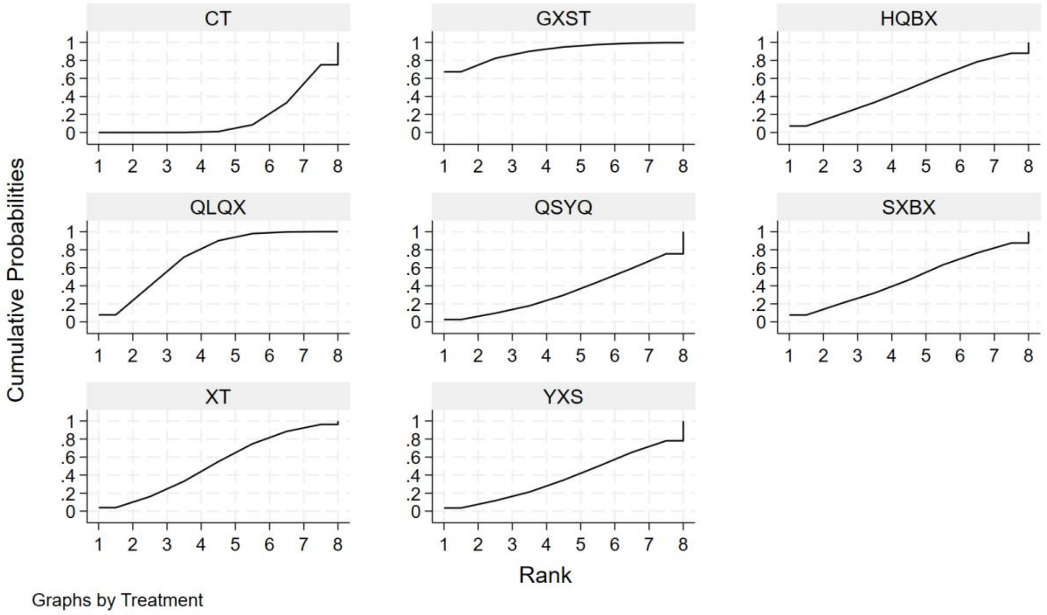

Supplementary Figure S7. SUCRA plots for 6MWT.

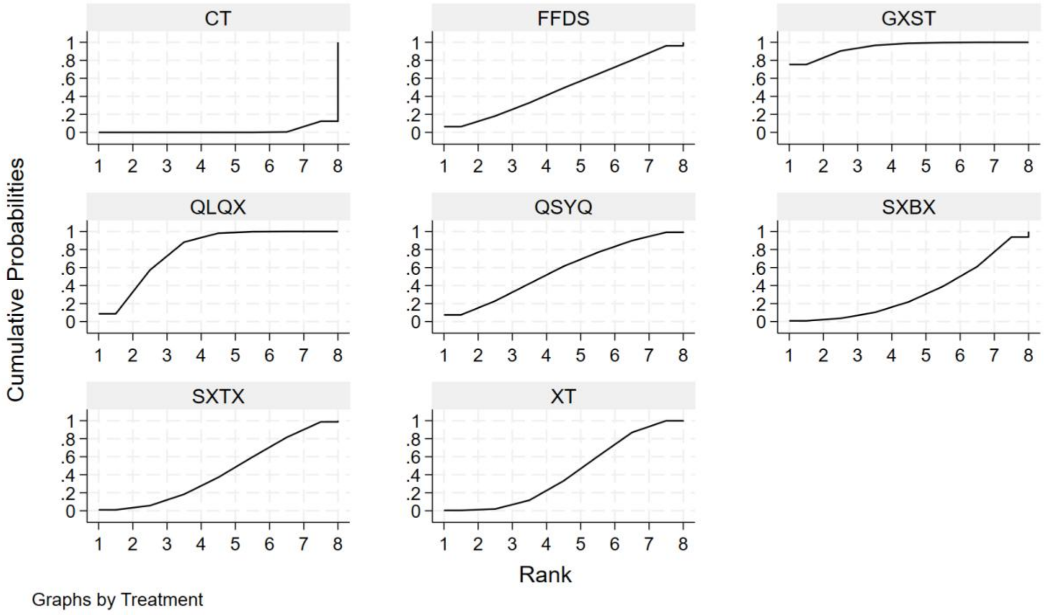

Supplementary Figure S8. Forest plot of the overall effect of oral CPMs combined with conventional treatment on LVEF.

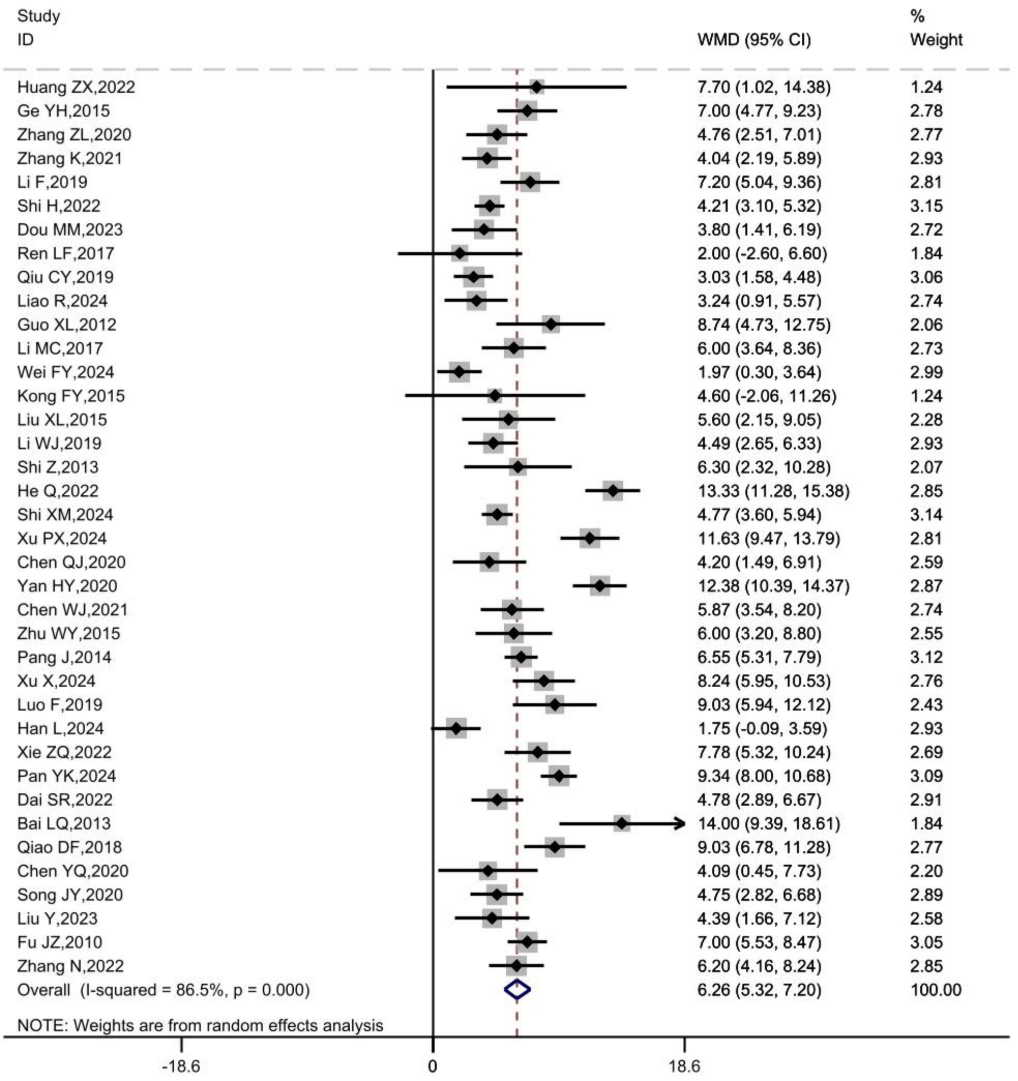

Note: The solid vertical line represents the line of no effect (WMD = 0). The diamonds at the bottom indicate the overall weighted mean difference (WMD) with 95% confidence intervals (CI) calculated using a random-effects model.

Supplementary Figure S9. Subgroup analysis of LVEF based on different CT.

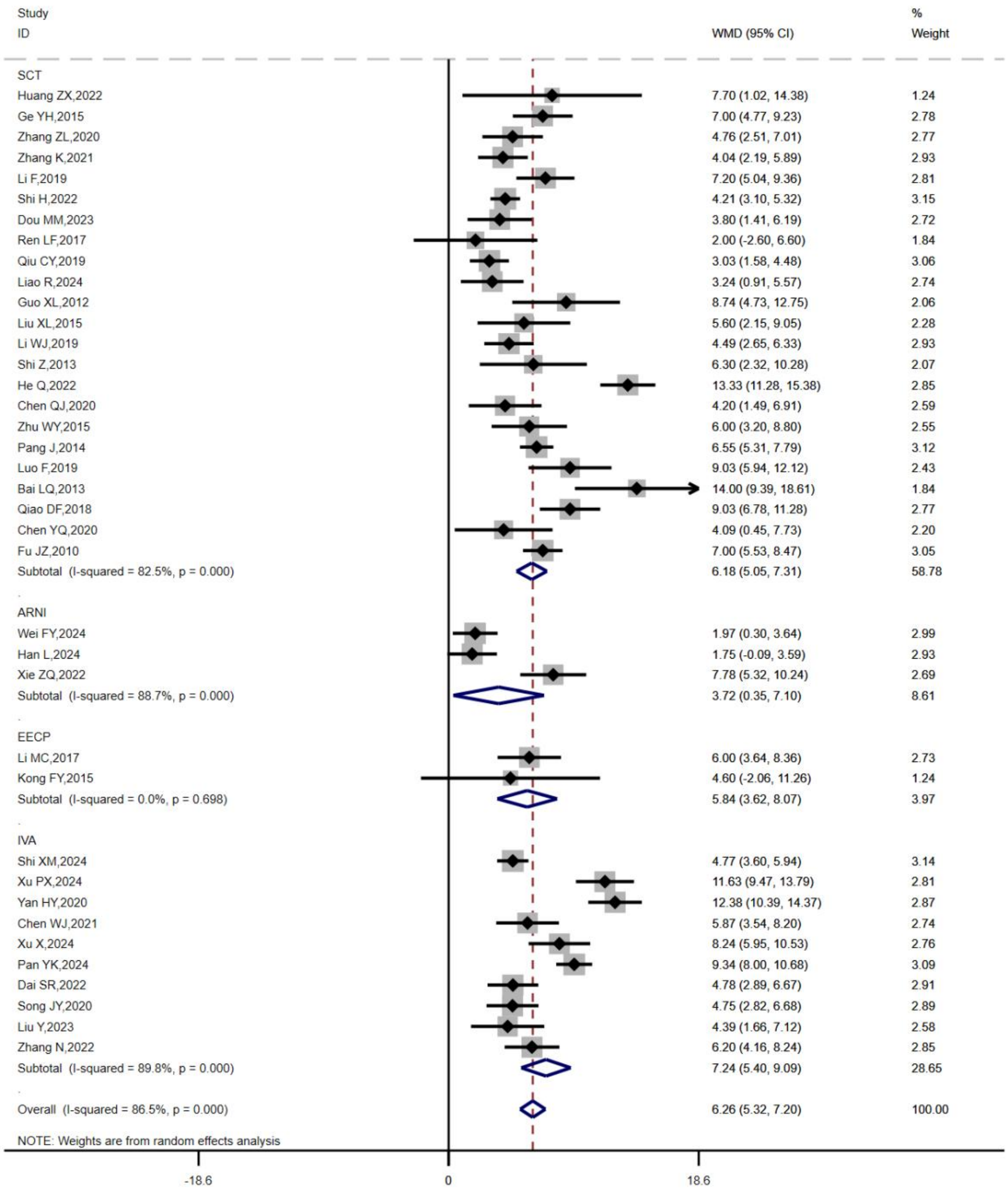

Note: The analysis categorizes the 42 included studies into four distinct subgroups according to the specific class and intensity of the control intervention: SCT (Standard Conventional Treatment), ARNI (Sacubitril/Valsartan), EECP (Enhanced External Counterpulsation), and IVA (Intravenous Vasoactive Agents). The pooled WMD for each subgroup confirms significant therapeutic benefits across all strategies without crossing the line of no effect. WMD, weighted mean difference; CI, confidence interval.

Supplementary Figure S10. Subgroup analysis of LVEF structured by the duration of combination therapy.

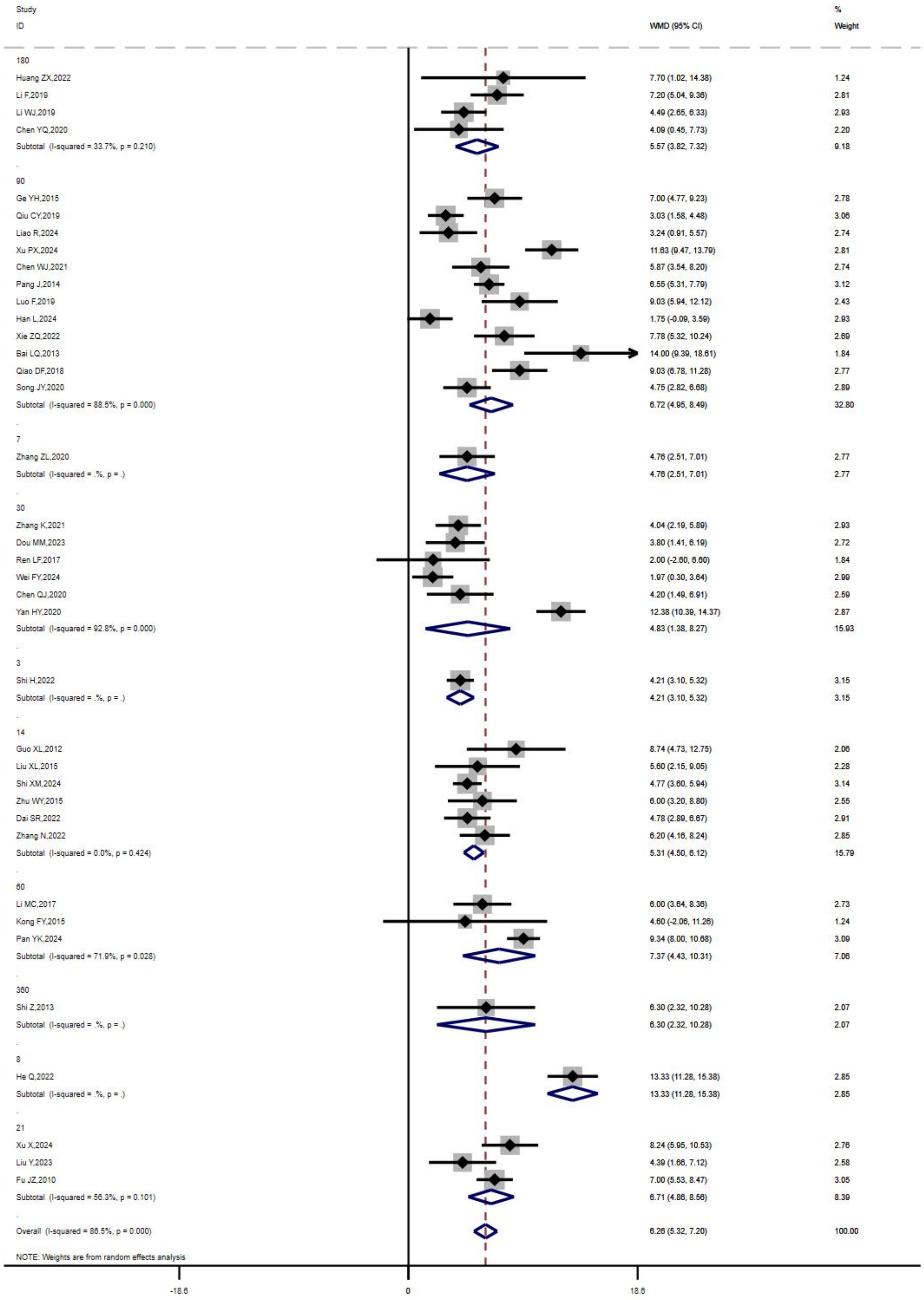

Note: The meta-analysis is stratified by treatment duration (ranging from 3 to 360 days) to explore temporal heterogeneity. The diamond symbols represent the pooled weighted mean difference (WMD) for each specific time course using a random-effects model. Notably, the 14-day subgroup demonstrates the highest consistency (I^2 = 0.0%). WMD, weighted mean difference; CI, confidence interval.

Supplementary Figure S11. Subgroup analysis of LVEF stratified by the stage of myocardial infarction(MI).

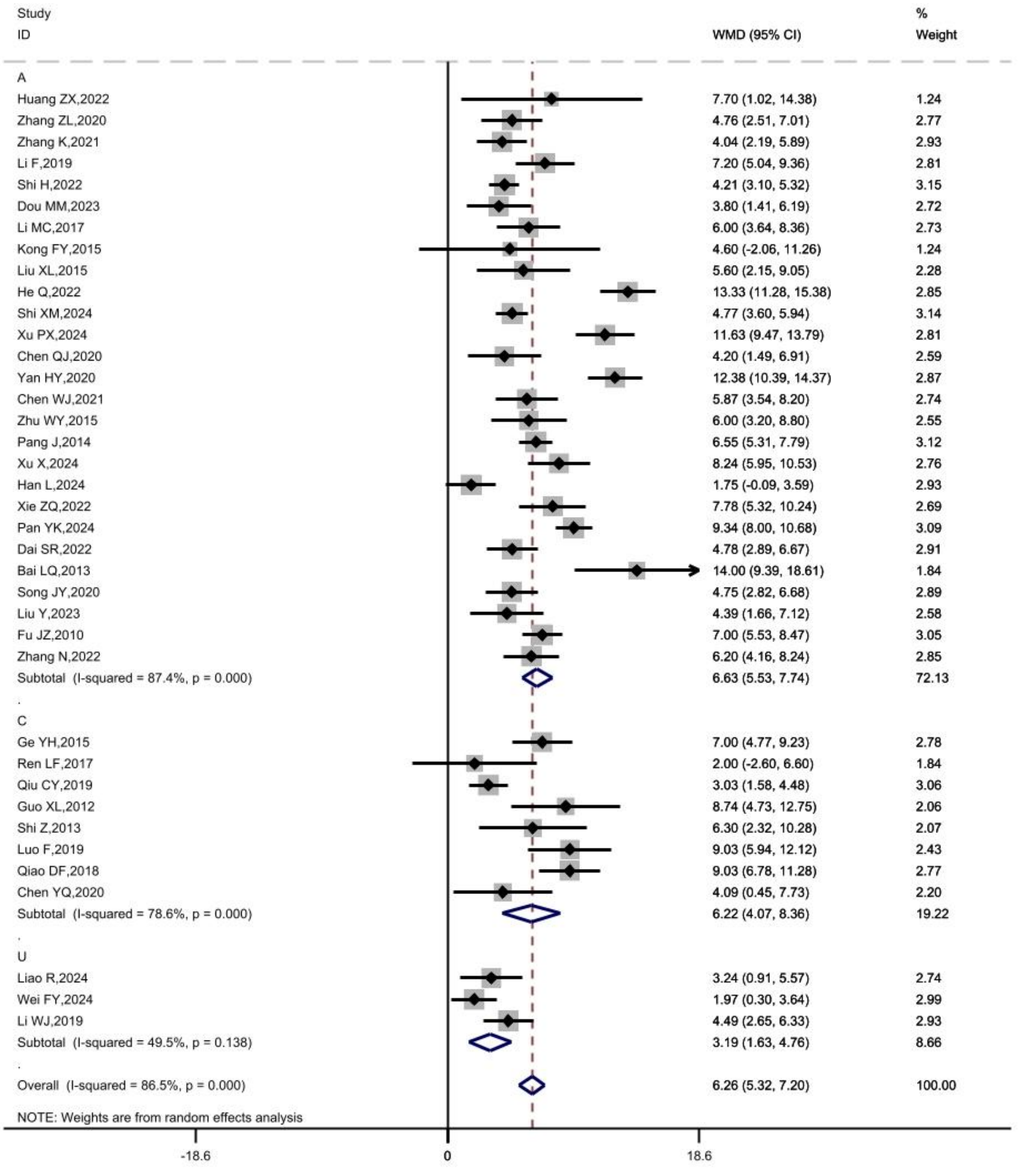

Note: Studies are categorized based on the phase of myocardial infarction at enrollment: A (Acute myocardial infarction), C (Chronic myocardial infarction), and U (Unspecified or mixed cohorts). The subgroup analysis confirms that the addition of oral Chinese patent medicines significantly improves LVEF across all documented disease stages. WMD, weighted mean difference; CI, confidence interval.

Supplementary Figure S12. Subgroup analysis of LVEF categorized by specific CPMs.

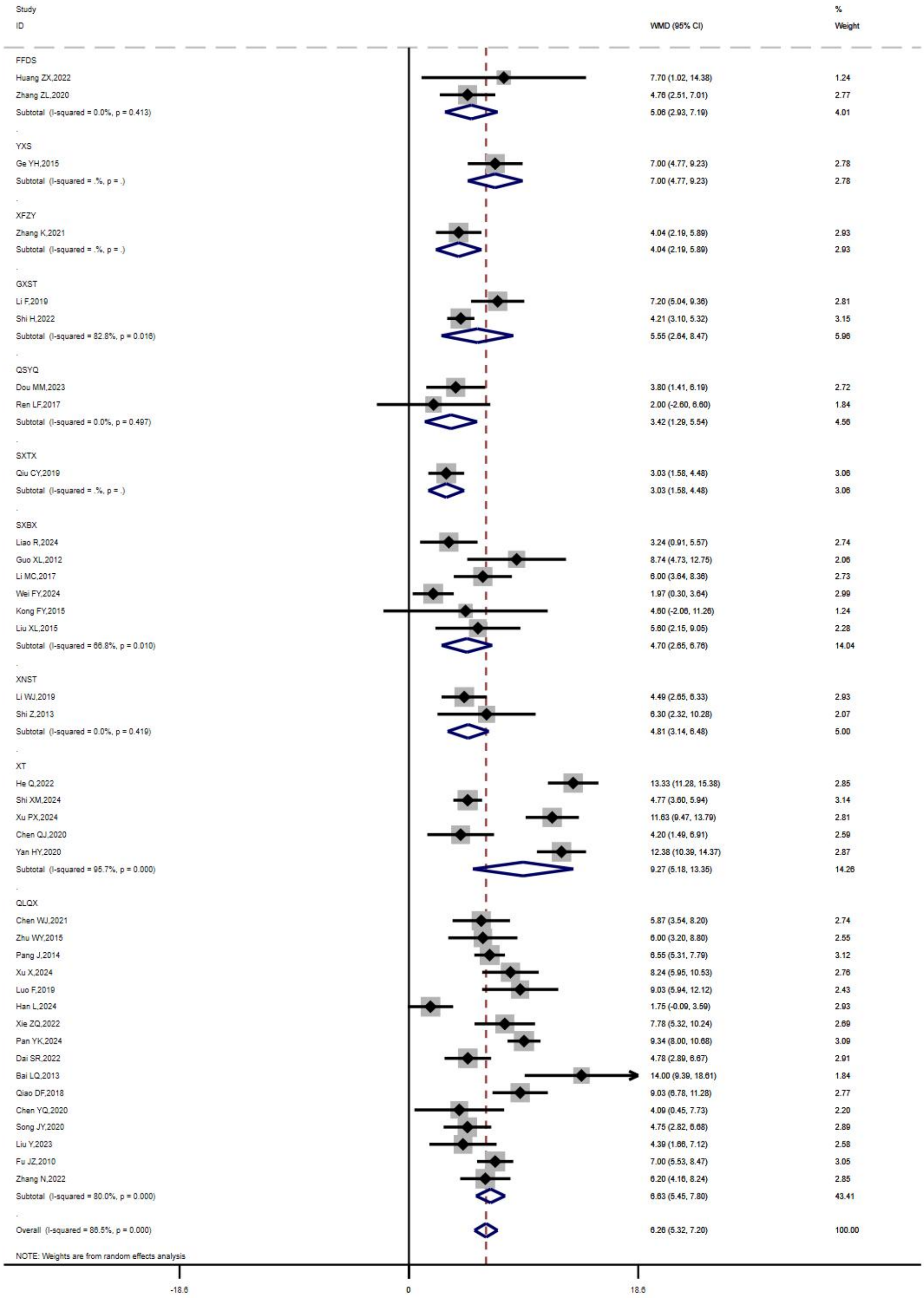

Note: The analysis illustrates the comparative efficacy of individual oral Chinese patent medicines against their respective control groups. Abbreviations: FFDS, Compound Danshen Dripping Pills; YXS, Yixinshu Capsules; XFZY, Xuefu Zhuyu Capsules; GXST, Guanxin Shutong Capsules; QSYQ, Qishen Yiqi Dripping Pills; SXTX, Shexiang Tongxin Dripping Pills; SXBX, Shexiang Baoxin Pills; XNST, Xinnao Shutong Capsules; XT, Xintong Oral Liquid; QLQX, Qili Qiangxin Capsules.

Supplementary Figure S13. Subgroup analysis of LVEDD categorized by specific CPMs.

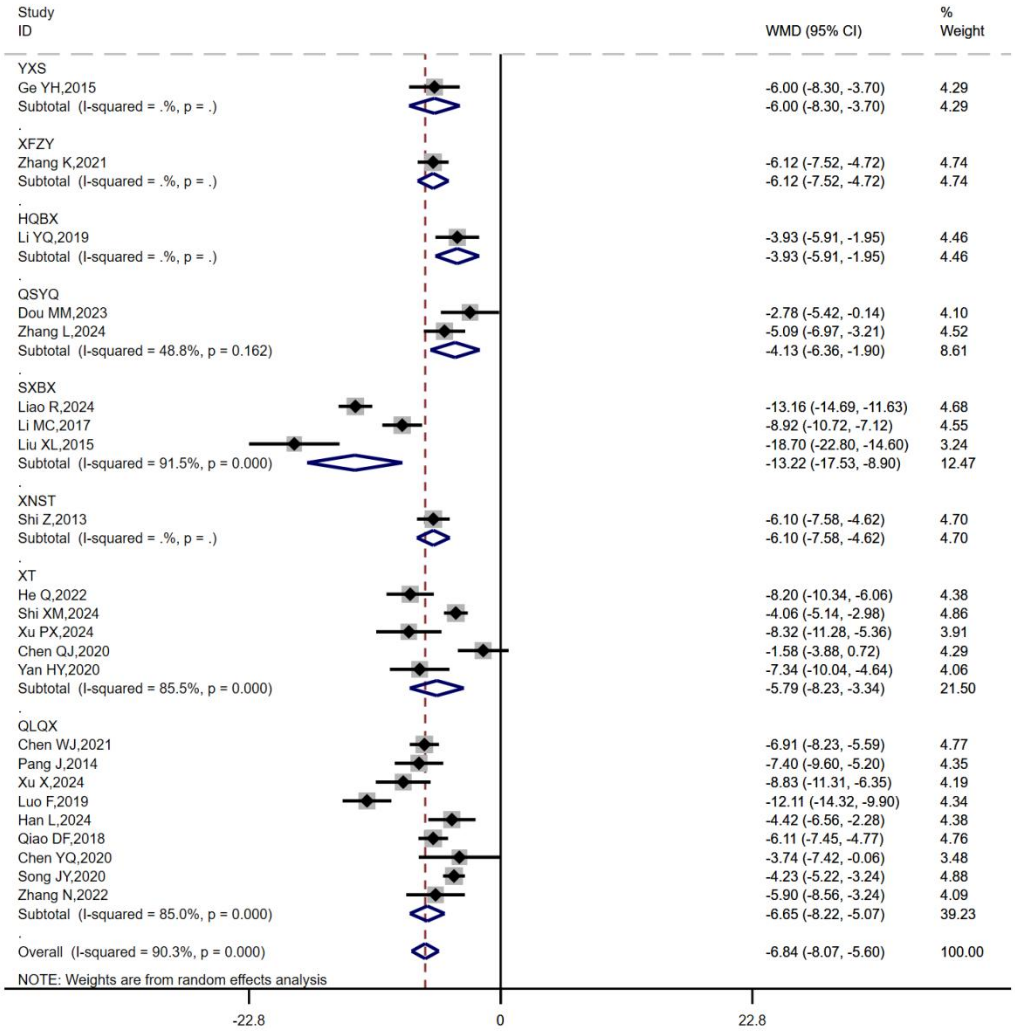

Note: The meta-analysis stratifies the effect on LVEDD reductions by the individual type of oral Chinese patent medicine investigated. The solid vertical line represents the line of no effect. Diamonds represent the pooled WMD for each subgroup evaluated using a random-effects model. Abbreviations: YXS, Yixinshu Capsules; XFZY, Xuefu Zhuyu Capsules; HQBX, Huangqi Baoxin Granules; QSYQ, Qishen Yiqi Dripping Pills; SXBX, Shexiang Baoxin Pills; XNST, Xinnao Shutong Capsules; XT, Xintong Oral Liquid; QLQX, Qili Qiangxin Capsules. WMD, weighted mean difference; CI, confidence interval.

Supplementary Figure S14. Subgroup analysis of LVEDD based on different CT background regimens.

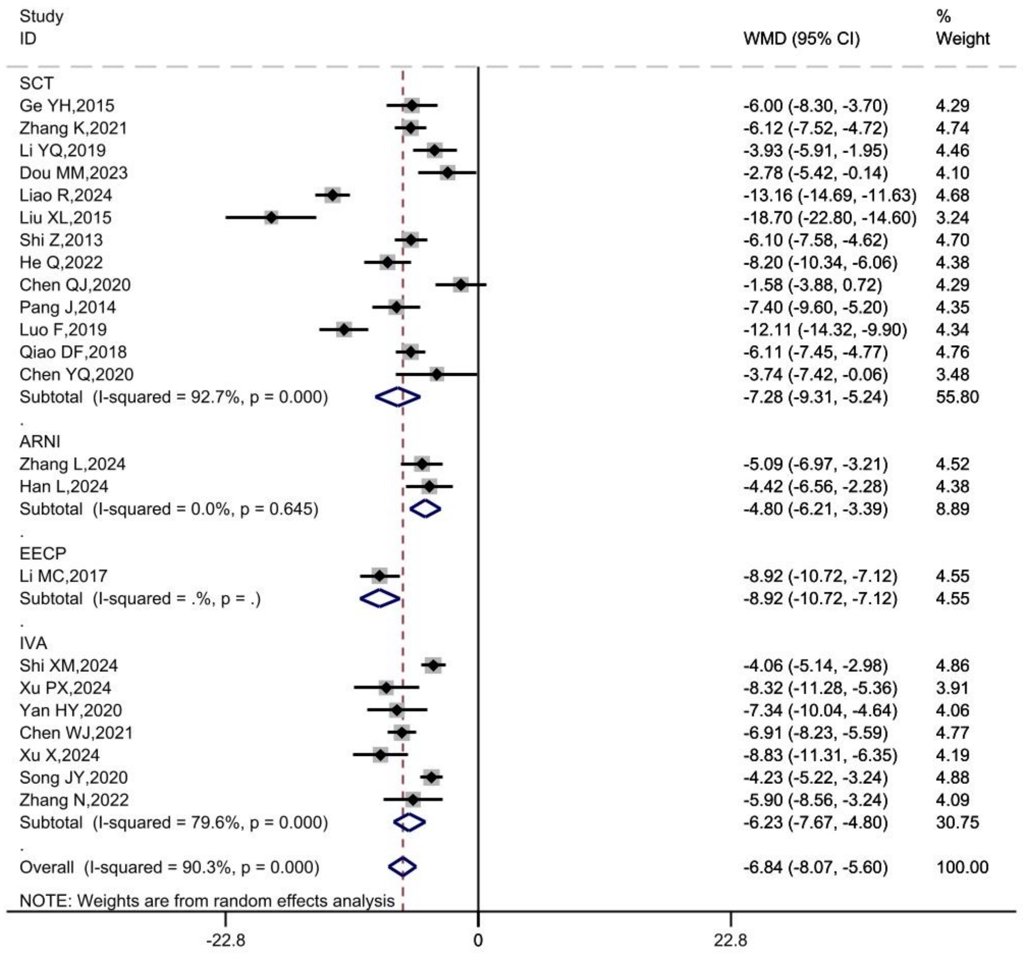

Note: The analysis categorizes the 23 included studies reporting LVEDD outcomes into four subgroups according to the specific class of control intervention: SCT (Standard Conventional Treatment), ARNI (Sacubitril/Valsartan), EECP (Device-Assisted Therapy), and IVA (Intravenous Vasoactive Agents). The pooled WMD for all subgroups falls to the left of the line of no effect, confirming significant therapeutic benefits across varied background strategies. Notably, stratification by ARNI fully resolved within-group heterogeneity (I^2 = 0.0%). WMD, weighted mean difference; CI, confidence interval.

Supplementary Figure S15. Subgroup analysis of LVESD based on different CT background regimens.

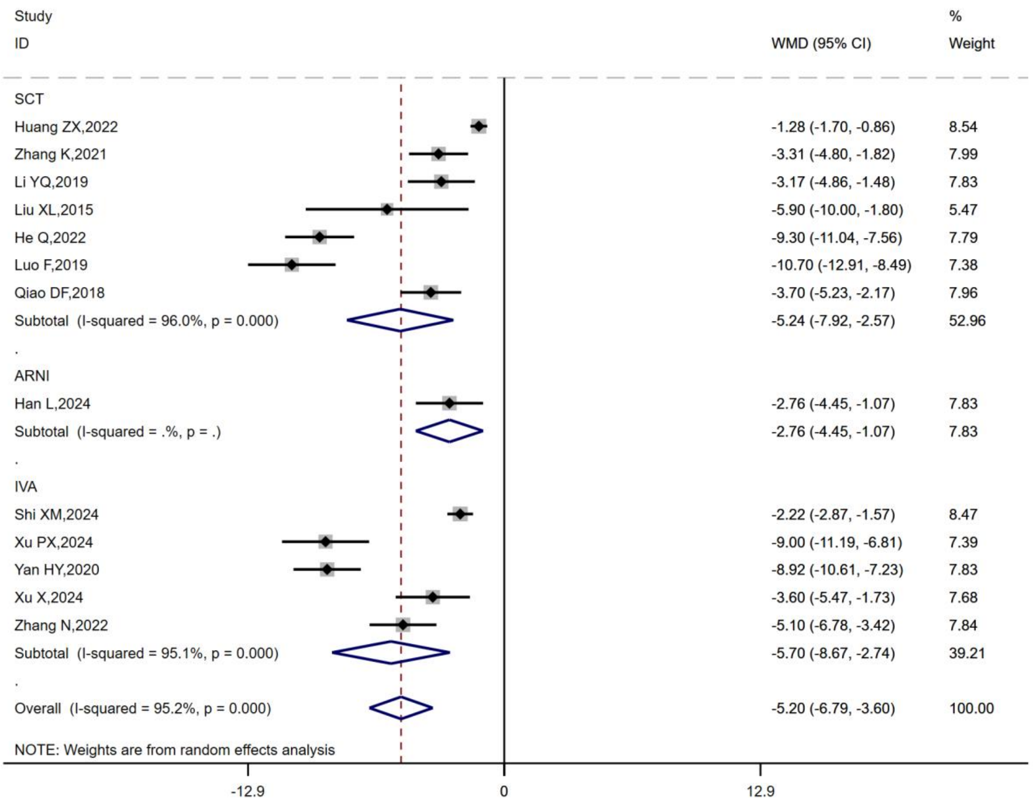

Note: The meta-analysis stratifies the effect on LVESD reductions according to the specific class of control interventions evaluated in the 13 included trials: SCT (Standard Conventional Treatment), ARNI (Sacubitril/Valsartan), and IVA (Intravenous Vasoactive Agents). The pooled WMD for all subgroups falls to the left of the vertical line of no effect, confirming significant and consistent reverse remodeling benefits across varied background strategies. WMD, weighted mean difference; CI, confidence interval.

Supplementary Figure S16. Subgroup analysis of LVESD categorized by specific CPMs.

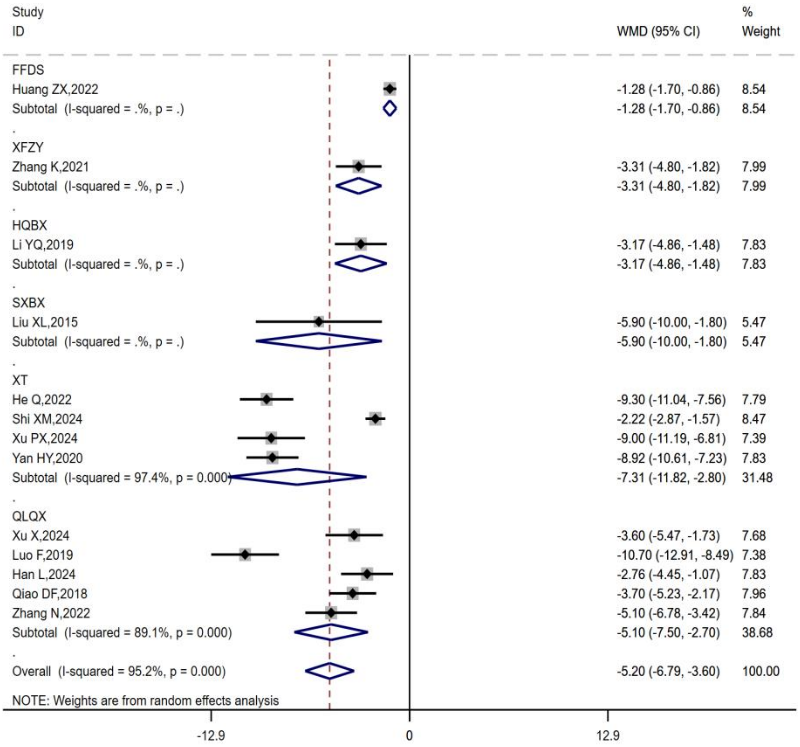

Note: The meta-analysis stratifies the therapeutic effect on LVESD according to the individual type of oral Chinese patent medicine investigated. Diamonds represent the pooled WMD for each subgroup evaluated using a random-effects model, comparing the respective CPM combined with conventional treatment against conventional treatment alone. WMD, weighted mean difference; CI, confidence interval.

Supplementary Figure S17. Subgroup analysis of NT-proBNP categorized by specific CPMs.

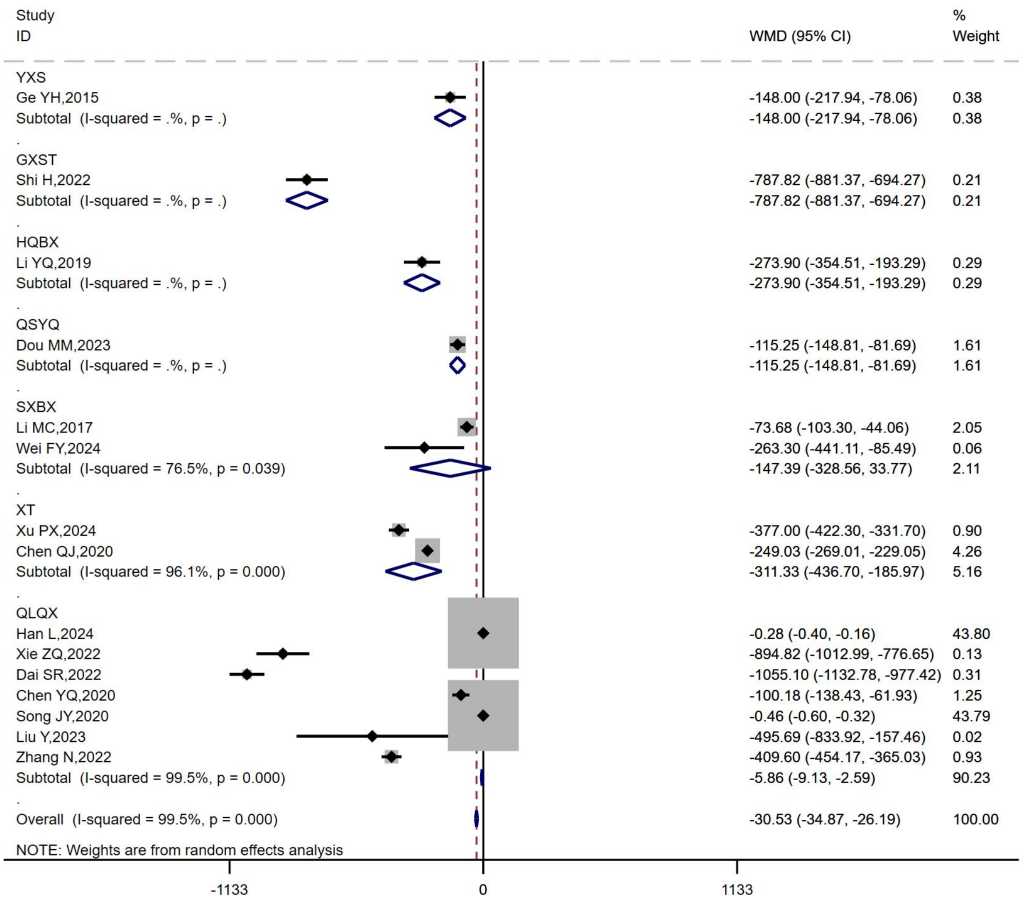

Supplementary Figure S18. Subgroup analysis of NT-proBNP stratified by the stage of MI.

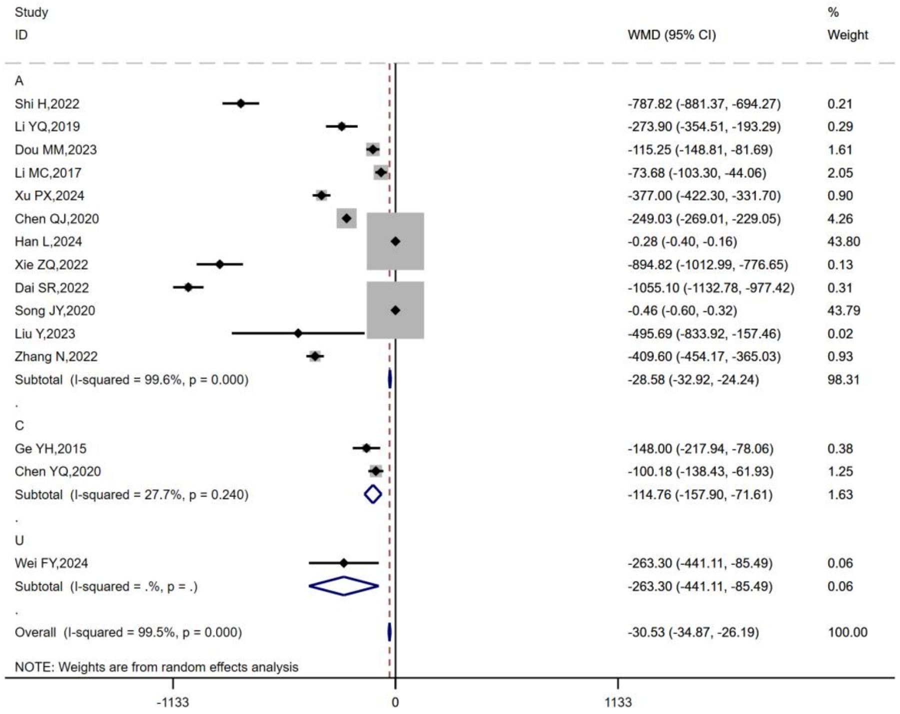

Note: Studies represent varying clinical phases of myocardial infarction. The subgroup analysis details the reduction of NT-proBNP among varying infarction stages, identifying temporal factors that influence therapeutic stability. SMD, standardized mean difference; CI, confidence interval.

Supplementary Figure S19. Subgroup analysis of NT-proBNP based on different CT background regimens.

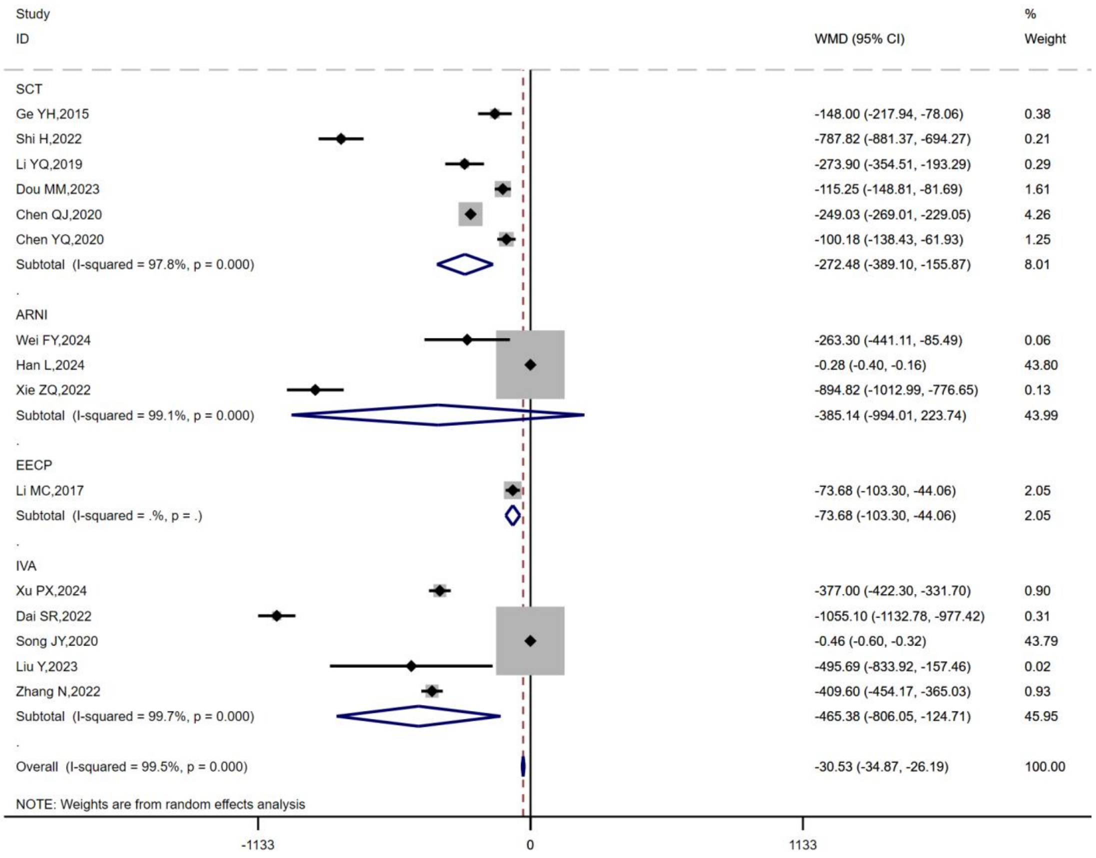

Note: The meta-analysis stratifies the effect on reducing NT-proBNP levels based on the intensity and class of background conventional therapies: SCT (Standard Conventional Treatment), ARNI (Sacubitril/Valsartan), EECP (Device-Assisted Therapy), and IVA (Intravenous Vasoactive Agents). The pooled SMD for the SCT, EECP, and IVA subgroups indicated significant reduction benefits. The ARNI subgroup exhibited a trend toward NT-proBNP reduction, though its confidence interval marginally crossed the line of no effect. SMD, standardized mean difference; CI, confidence interval.

Supplementary Figure S20. Subgroup analysis of the 6MWT distance categorized by specific CPMs.

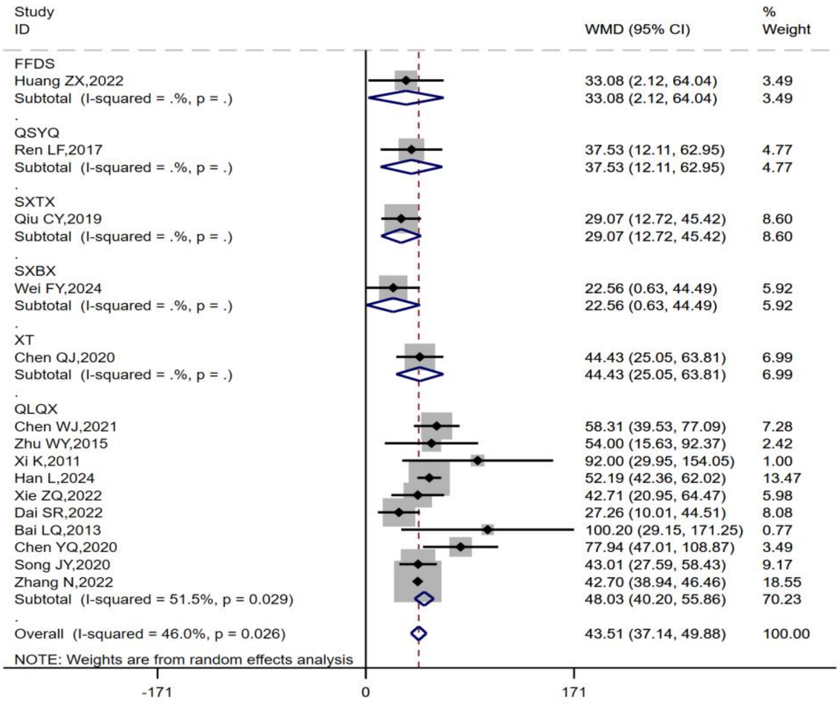

Note: The meta-analysis illustrates the comparative efficacy in extending the 6MWT distance when separated by the individual type of oral Chinese patent medicine prescribed. WMD, weighted mean difference; CI, confidence interval.

Supplementary Figure S21. Subgroup analysis of the 6MWT distance stratified by the stage of MI.

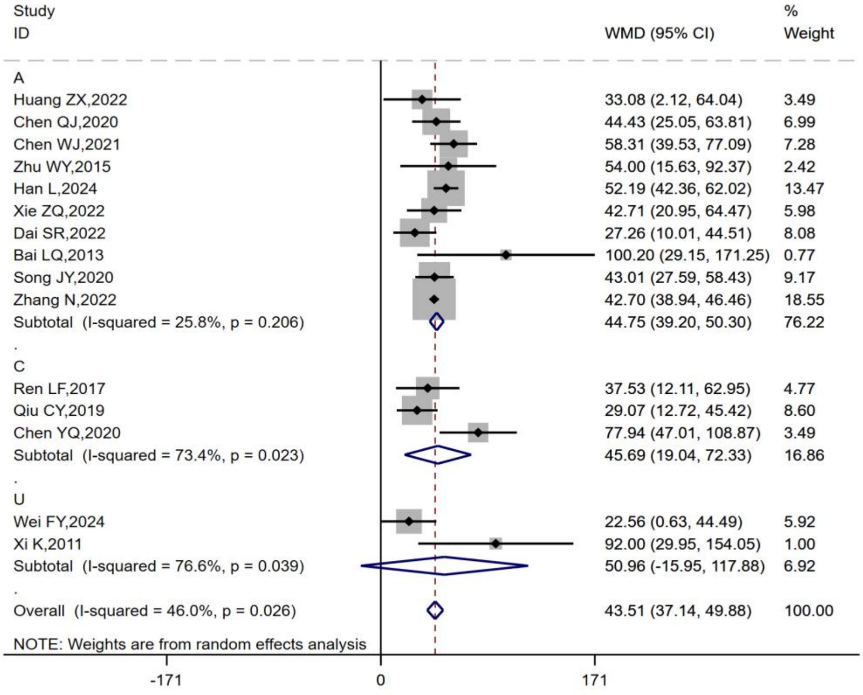

Note: Studies are categorized based on whether patients presented with acute or chronic/old myocardial infarction to test temporal robustness. WMD, weighted mean difference; CI, confidence interval.

Supplementary Figure S22. Subgroup analysis of the 6MWT distance structured by the duration of combination therapy.

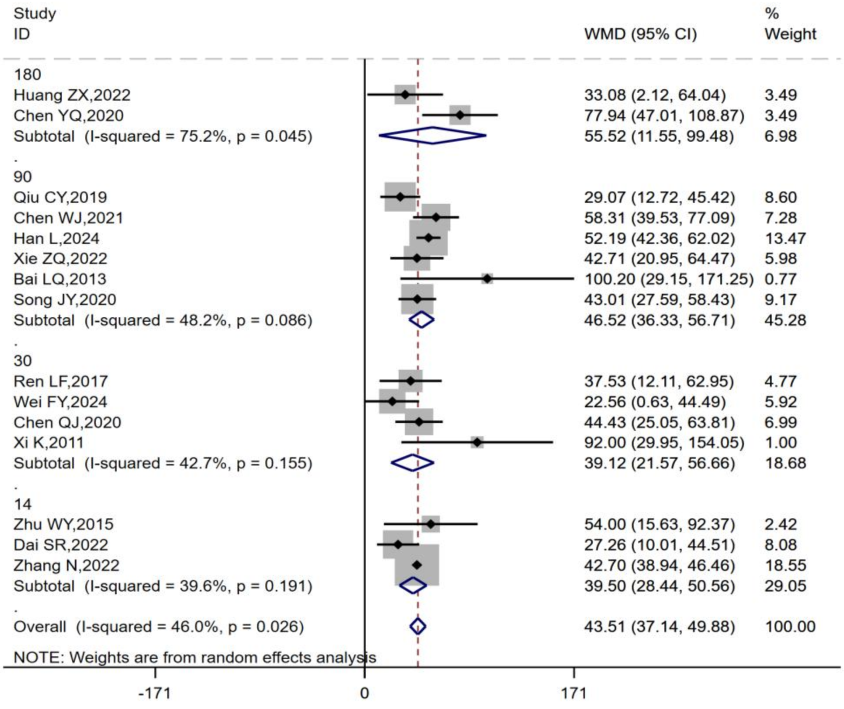

Note: The analysis details the improvement in exercise tolerance across varying treatment durations, indicating optimal therapeutic windows. WMD, weighted mean difference; CI, confidence interval.

Supplementary Figure S23. Subgroup analysis of the 6MWT distance based on different CT background regimens.

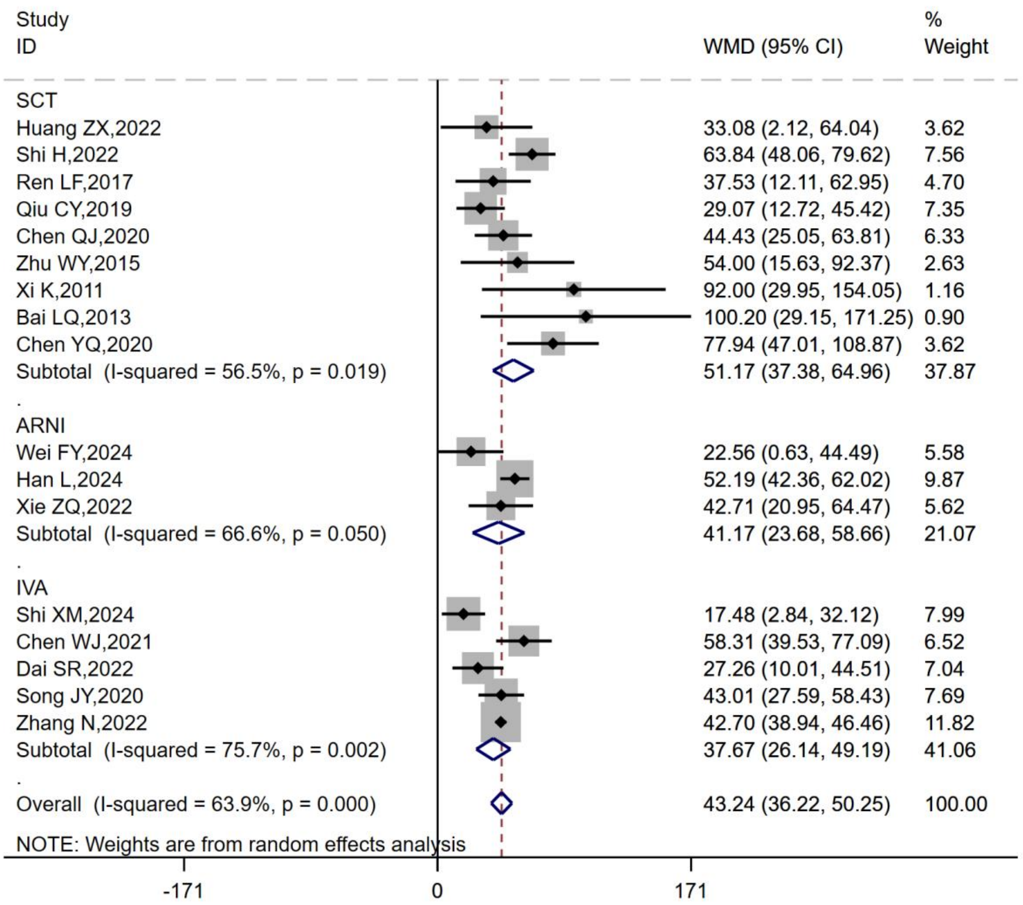

Note: The meta-analysis stratifies the therapeutic improvements in 6MWT distance according to the intensity and class of background conventional therapies: SCT (Standard Conventional Treatment), ARNI (Sacubitril/Valsartan), and IVA (Intravenous Vasoactive Agents). The pooled WMD for all subgroups falls decisively to the right of the line of no effect, confirming that the addition of Chinese patent medicines significantly and consistently enhances exercise tolerance regardless of the baseline CWM regimen. WMD, weighted mean difference; CI, confidence interval.

Appendix S1 Search strategy

1.Pubmed

(((("Medicine, Chinese Traditional"[Mesh]) OR ((((((((((Medicine, Chinese Traditional[Title/Abstract]) OR (Capsules[Title/Abstract])) OR (granules[Title/Abstract])) OR (powders[Title/Abstract])) OR (Tablets[Title/Abstract])) OR (Drug Implants[Title/Abstract])) OR (Drug Pellets[Title/Abstract])) OR (oral liquid[Title/Abstract])) OR (Unguentum[Title/Abstract])) OR (Chinese patent drugs[Title/Abstract]))) AND (acute[Title/Abstract])) AND (("Myocardial Infarction"[Mesh]) OR ((((((((((((((myocardial infarction[Title/Abstract]) OR (Infarction, Myocardial[Title/Abstract])) OR (Infarctions, Myocardial[Title/Abstract])) OR (Myocardial Infarctions[Title/Abstract])) OR (Heart Attack[Title/Abstract])) OR (Heart Attacks[Title/Abstract])) OR (Myocardial Infarct[Title/Abstract])) OR (Infarct, Myocardial[Title/Abstract])) OR (Infarcts, Myocardial[Title/Abstract])) OR (Myocardial Infarcts[Title/Abstract])) OR (Cardiovascular Stroke[Title/Abstract])) OR (Cardiovascular Strokes[Title/Abstract])) OR (Stroke, Cardiovascular[Title/Abstract])) OR (Strokes, Cardiovascular[Title/Abstract])))) AND (("Heart Failure"[Mesh]) OR (((((((((((((((heart failure[Title/Abstract]) OR (Cardiac Failure[Title/Abstract])) OR (Heart Decompensation[Title/Abstract])) OR (Decompensation, Heart[Title/Abstract])) OR (Congestive Heart Failure[Title/Abstract])) OR (Heart Failure, Congestive[Title/Abstract])) OR (Heart Failure, Right-Sided[Title/Abstract])) OR (Heart Failure, Right Sided[Title/Abstract])) OR (Right-Sided Heart Failure[Title/Abstract])) OR (Right Sided Heart Failure[Title/Abstract])) OR (Heart Failure, Left-Sided[Title/Abstract])) OR (Heart Failure, Left Sided[Title/Abstract])) OR (Left-Sided Heart Failure[Title/Abstract])) OR (Left Sided Heart Failure[Title/Abstract])) OR (Myocardial Failure[Title/Abstract])))

2.cochrane library

#1 MeSH descriptor: [Medicine, Chinese Traditional] explode all trees

#2 Medicine, Chinese Tradtional OR Capsules OR granules OR powders OR Tablets OR Drug Implants OR Drug Pellets OR oral liquid OR Unguentum OR Chinese patent drugs OR Traditional Medicine, Chinese OR Chinese Traditional Medicine OR Traditional Chinese Medicine

#3 #1 OR #2

#4 MeSH descriptor: [Myocardial Infarction] explode all trees

#5 Myocardial Infarction OR Infarcts, Myocardial OR Heart Attacks OR Stroke, Cardiovascular OR Infarct, Myocardial OR Cardiovascular Strokes OR Infarction, Myocardial OR Myocardial Infarct OR Strokes, Cardiovascular OR Infarctions, Myocardial OR Myocardial Infarcts OR Myocardial Infarctions OR Heart Attack OR Cardiovascular Stroke

#6 #4 OR #5

#7 MeSH descriptor: [Heart Failure] explode all trees

#8 Heart Failure, Congestive OR Congestive Heart Failure OR Cardiac Failure OR Right-Sided Heart Failure OR Heart Failure, Right Sided OR Heart Failure, Right-Sided OR Right Sided Heart Failure OR Heart Failure, Left Sided OR Heart Failure, Left-Sided OR Left-Sided Heart Failure OR Left Sided Heart Failure OR Myocardial Failure OR Heart Decompensation OR Decompensation, Heart

#9 #7 OR #8

#10 #3 AND #6 AND #9

3.web of science

#1 TS= (Medicine, Chinese Traditional OR Medicine, Chinese Traditional OR Capsules OR granules OR powders OR Tablets OR Drug Implants OR Drug Pellets OR oral liquid OR Unguentum OR Chinese patent drugs)

#2 TS= (Myocardial infarction OR Infarction, Myocardial OR Infarctions, Myocardial OR Myocardial Infarctions OR Heart Attack OR Heart Attacks OR Myocardial Infarct OR Infarct, Myocardial OR Infarcts, Myocardial OR Myocardial Infarcts OR Cardiovascular Stroke OR Cardiovascular Strokes OR Stroke, Cardiovascular OR Strokes, Cardiovascular)

#3 TS= (Heart failure OR Cardiac Failure OR Heart Decompensation OR Decompensation, Heart OR Congestive Heart Failure OR Heart Failure, Congestive OR Heart Failure, Right-Sided OR Heart Failure, Right Sided OR Right-Sided Heart Failure OR Right Sided Heart Failure OR Heart Failure, Left-Sided OR Heart Failure, Left Sided OR Left-Sided Heart Failure OR Left Sided Heart Failure OR Myocardial Failure)

#4 #1 AND #2 AND #3

4.embase

#1 'chinese medicine'/exp OR 'chinese medicine'

#2 'chinese traditional medicine':ab,ti OR 'medicine, chinese traditional':ab,ti OR 'traditional chinese medicine':ab,ti OR 'chinese medicine':ab,ti OR capsules:ab,ti OR granules:ab,ti OR powders:ab,ti OR tablets:ab,ti OR 'oral liquid':ab,ti OR 'chinese

patent drugs':ab,ti

#3 #1 OR #2

#4 'heart infarction'/exp

#5 'cardiac infarct':ab,ti OR 'cardiac infarction':ab,ti OR 'cardial infarct':ab,ti OR 'heart attack':ab,ti OR 'heart infarct':ab,ti OR 'heart micro infarction':ab,ti OR 'heart muscle infarction':ab,ti OR 'infarction, heart':ab,ti OR 'myocardial infarct':ab,ti OR 'myocardial infarction':ab,ti OR 'myocardium infarct':ab,ti OR 'myocardium infarction':ab,ti OR 'premonitory infarction sign':ab,ti OR 'second heart attack':ab,ti OR 'subendocardial infarction':ab,ti OR 'transmural cardiac infarction':ab,ti OR 'transmural heart infarction':ab,ti OR 'transmural infarction, heart':ab,ti OR 'heart infarction':ab,ti

#6 #4 OR #5

#7 'heart failure'/exp

#8 'backward failure, heart':ab,ti OR 'cardiac backward failure':ab,ti OR 'cardiac decompensation':ab,ti OR 'cardiac failure':ab,ti OR 'cardiac incompetence':ab,ti OR 'cardiac insufficiency':ab,ti OR 'cardiac stand still':ab,ti OR 'cardial decompensation':ab,ti OR 'cardial insufficiency':ab,ti OR 'chronic heart failure':ab,ti OR 'chronic heart insufficiency':ab,ti OR 'decompensatio cordis':ab,ti OR 'decompensation, heart':ab,ti OR 'heart backward failure':ab,ti OR 'heart decompensation':ab,ti OR 'heart incompetence':ab,ti OR 'heart insufficiency':ab,ti OR 'insufficientia cardis':ab,ti OR 'myocardial failure':ab,ti OR 'myocardial insufficiency':ab,ti OR 'heart failure':ab,ti

#9 #7 OR #8

#10 'randomized controlled trial'/exp OR 'randomized controlled trial'

#11 'controlled trial, randomized':ab,ti OR 'randomised controlled study':ab,ti OR 'randomised controlled trial':ab,ti OR 'randomized controlled study':ab,ti OR 'trial, randomized controlled':ab,ti OR 'randomized controlled trial':ab,ti

#12 #10 OR #11

#13 #3 AND #6 AND #9 AND #12
